# Interplay between Constraints, Objectives, and Optimality for Genome-Scale Stoichiometric Models

**DOI:** 10.1371/journal.pcbi.1004166

**Published:** 2015-04-07

**Authors:** Timo R. Maarleveld, Meike T. Wortel, Brett G. Olivier, Bas Teusink, Frank J. Bruggeman

**Affiliations:** 1 Systems Bioinformatics, Amsterdam Institute for Molecules, Medicines and Systems, VU University, Amsterdam, The Netherlands; 2 Life Sciences, Centrum Wiskunde & Informatica (CWI), Amsterdam, The Netherlands; 3 Kluyver Centre for Genomics of Industrial Fermentation, Delft, The Netherlands; Chalmers University of Technology, SWEDEN

## Abstract

High-throughput data generation and genome-scale stoichiometric models have greatly facilitated the comprehensive study of metabolic networks. The computation of all feasible metabolic routes with these models, given stoichiometric, thermodynamic, and steady-state constraints, provides important insights into the metabolic capacities of a cell. How the feasible metabolic routes emerge from the interplay between flux constraints, optimality objectives, and the entire metabolic network of a cell is, however, only partially understood. We show how optimal metabolic routes, resulting from flux balance analysis computations, arise out of elementary flux modes, constraints, and optimization objectives. We illustrate our findings with a genome-scale stoichiometric model of *Escherichia coli* metabolism. In the case of one flux constraint, all feasible optimal flux routes can be derived from elementary flux modes alone. We found up to 120 million of such optimal elementary flux modes. We introduce a new computational method to compute the corner points of the optimal solution space fast and efficiently. Optimal flux routes no longer depend exclusively on elementary flux modes when we impose additional constraints; new optimal metabolic routes arise out of combinations of elementary flux modes. The solution space of feasible metabolic routes shrinks enormously when additional objectives---e.g. those related to pathway expression costs or pathway length---are introduced. In many cases, only a single metabolic route remains that is both feasible and optimal. This paper contributes to reaching a complete topological understanding of the metabolic capacity of organisms in terms of metabolic flux routes, one that is most natural to biochemists and biotechnologists studying and engineering metabolism.

## Author Summary

Organisms depend on huge networks of molecular reactions for environmental sensing, information integration, gene expression, and metabolism. The discovery of general principles of network behavior is a major ambition of systems biology and of great interest to biotechnology and medicine. We present a computational tool that calculates all optimal states of metabolism in terms of pathways, which is arguably the most intuitive and preferred approach to characterize whole-cell metabolism. We show how the space of all feasible flux distributions can be compactly described in terms of a unique set of minimal and feasible pathways, given realistic stoichiometric, thermodynamic, and optimization-objective constraints. This description clarifies the interplay between flux constraints and optimization objectives. We explain why some fluxes are variable and cross-correlate within the solution space while others do not and how multi-objective optimization shrinks the solution space. We illustrate our findings with a toy metabolic model to explain the main insights and apply it to a genome-scale stoichiometric model of *Escherichia coli* metabolism.

## Introduction

Research in biotechnology and medicine benefits from understanding the metabolic capacity of organisms, including their sensitivities to genetic and environmental changes. Genome-scale stoichiometric models of metabolism [1, 2] and the availability of annotated genome sequences have greatly accelerated metabolic research. The combined use of high-throughput metabolomics data, comprehensive protocols [3], and (automated) reconstruction tools [4] has resulted in an explosion in the number and size of genome-scale stoichiometric metabolic models [5, 6]. Constraint-based modeling has become an indispensable tool to deal with these large models, used in biotechnology [7, 8] and medicine [9, 10].

The most common constraint-based modeling method is Flux Balance Analysis (FBA) [11, 12], which—given certain capacity constraints on fluxes—optimizes an objective function, e.g. the biomass production flux [13]. The accuracy of FBA predictions depends on the availability of realistic flux constraints, which can be derived from experimental data. Generally, there are insufficient flux constraints to obtain a single unique solution and a large space of optimal flux distributions results [14–16]. These alternative flux distributions give an impression of the robustness of a metabolic network [17], but not every alternative is equally favorable for the organism. In some environments organisms are strongly selected for yield, almost regardless of the protein burden, while in other environments the protein burden has a significant impact. The solution space can be analyzed further with secondary objectives [18–22], e.g. minimization of the number of active fluxes [23] or the sum of absolute fluxes [24], which have been used as proxies for maximization of the protein expression efficiency and minimization of the protein burden, respectively.

Analyzing the solution space and optimizing secondary objectives requires adequate mathematical and computations methods. Several approaches were proposed to give insight into the geometry of the optimal solution space [14, 15, 25–28], which is mathematically represented by a polyhedron [29]. Flux Variability Analysis (FVA) [14] and Flux Coupling Analysis (FCA) [25] provide valuable information on the boundaries of the solution space, but do not give understanding in terms of metabolic routes. Such an understanding would be extremely helpful, as most biologists intuitively think in terms of metabolic routes.

**Fig 1:**
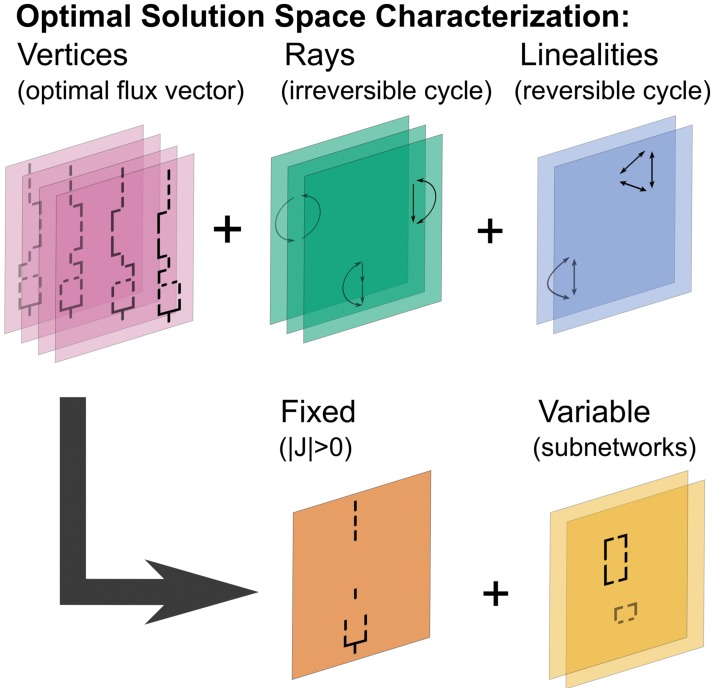
Characterization of the optimal solution space of metabolic models. The optimal solution space can be characterized by three topological features: vertices (purple), rays (green), and linealities (blue). Typically, optimal solution spaces of microbial genome-scale models are characterized by many vertices and only a few linealities and rays. Linealities do not exist when reversible reaction are split. Vertices can be described by a fixed active part (red) which is identical for each vertex and a variable part (orange), a few CoPE-FBA subnetworks [16]. We refer to S1 Fig. for examples of rays we found in the *E.coli* iAF1260 genome-scale metabolic model.

Characterization of the optimal solution space provides valuable insight into how our limited knowledge of constraints affects the prediction of a metabolic state of an organism. The recently developed method, CoPE-FBA (Comprehensive Polyhedron Enumeration FBA) [16], enumerates the vertices, the corner points of the optimal solution space. The number of vertices originates from the feasible, alternative metabolic routes through a small number of subnetworks, consisting only of reactions with correlated flux variability (Fig. 1). This method provides the structural insights that FVA and FCA lack, and explains the typical combinatorial explosion of the vertices; the optimal solution space can easily have millions of vertices that arise from independent combinations of alternative flux routes through only a few, small segments of the metabolic network. However, CoPE-FBA suffers from computational difficulties, it is slow, and—perhaps more important—the provided solution does not yield all non-decomposable flux routes in the optimum, limiting the use of CoPE-FBA. For instance, it cannot be used to assess the influence of secondary objectives on the solution space.

We aim to obtain a better understanding of the interplay between constraints, objectives, and optimality for genome-scale stoichiometric models. We uniquely characterize the optimal solution space by adjusting CoPE-FBA to split each reversible reaction into two irreversible reactions; this yields all non-decomposable flux routes in the optimum. We start by illustrating the differences between the CoPE-FBA outcomes of metabolic models with and without reversible-reaction splitting. Next, we explain the relationship between these vertices and elementary flux modes (EFMs) with an optimal substrate-product yield. Finally, we show that secondary objectives typically collapse the optimal solution space to a unique solution (a vertex) or to a small set of vertices, using the iAF1260 genome-scale model of *Escherichia coli* metabolism. Enumerating all non-decomposable flux routes in the optimum requires a more efficient computational method, which we also present in the Methods section of this work. This results in CoPE-FBA 2.0, our tool of choice for analyzing the optimal solution space in terms of network topology.

## Results

### Characterization of the optimal solution space: Illustration with a toy model

**Fig 2:**
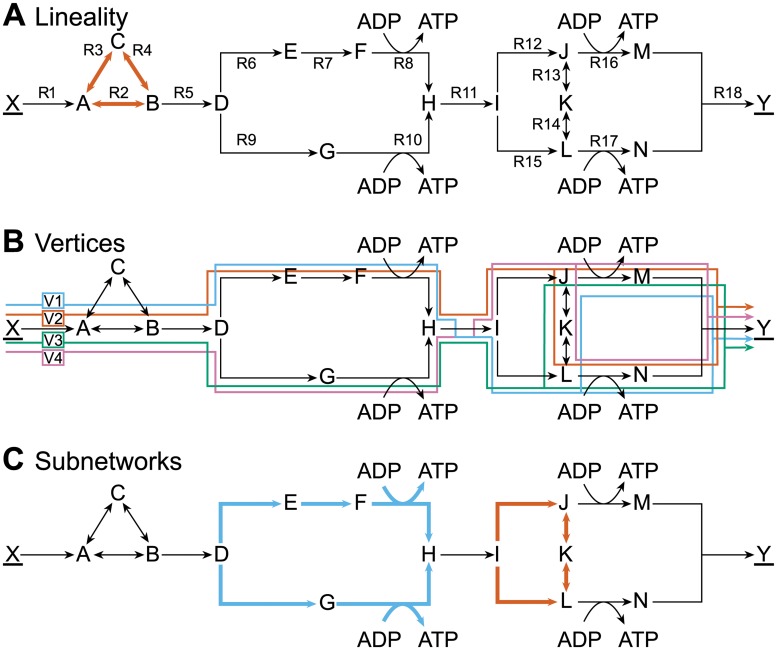
The optimal solution space of a toy model with reversible reactions. Metabolites (capital letters) are converted by reversible (two headed arrows) and irreversible (single headed arrows) reactions to achieve the conversion of X to Y (underlined metabolites are boundary species). The forward direction of reversible reactions is defined from left to right or from top to bottom, and a backwards flux is denoted by a minor sign (e.g. -R13 indicates conversion from K to J). We maximized the flux through R18 with FBA, subject to steady-state constraints and *J*_1_ ≤ 2, where *J*_1_ is the flux through reaction R1. The optimal solution space is characterized by (A) one lineality of reactions {R2–R4} (red) and (B) four vertices that arise from two branches at intersections D and I: V1 (blue), V2 (red), V3 (green) and V4 (purple) (C) Two CoPE-FBA subnetworks illustrate the alternatives that create the four vertices shown in (B); in subnetwork one (blue) these are {R6–R8} (V1 and V2) and {R9–R10} (V3 and V4), and in subnetwork two (red) {R12–R14} (V2 and V4) and {R15, -R13, -R14} (V1 and V3).

We developed the toy network shown in Fig. 2A to illustrate: (i) the characterization of the optimal solution space of an FBA in terms of metabolic flux routes, (ii) that reversible-reaction splitting guarantees finding all non-decomposable metabolic flux routes in the optimum, (iii) the relationship between vertices and optimal-yield EFMs, and (iv) the optimization of secondary objectives over the optimal solution space. Our toy network consists of 18 metabolites and reactions where the source metabolite X and sink metabolite Y are considered boundary metabolites. All reactions, besides the reactions where ATP and ADP act as cofactors, are isomerization (uni-uni) reactions and reversible reactions are illustrated by two headed arrows.

For our FBA model, we selected maximization of the flux through reaction R18 as our objective function, *Z*_*obj*_. To constrain the solution space we used one inequality constraint, *J*_1_ ≤ 2. Throughout this work, we call this type of (inequality) constraint a restricting non-zero flux constraint. The resulting FBA is formulated as the linear program:

$$\begin{array}{ccc} & & \text{Maximize}\;Z_{obj}=J_{18}\\ & & \text{subject}\;\text{to,}\\ & & \;\;\;\;\;N\;J=0\\ & & \;\;\;\;\;-\infty \leq J_{r}\leq \infty \;\;\;\;\;\;J_{r}\in \text{reversible}\;\text{reactions}\\ & & \;\;\;\;\;\;0\leq J_{i}\leq \infty \;\;\;\;\;\;\;\;\;\;J_{i}\in \text{irreversible}\;\text{reactions}\\ & & \;\;\;\;\;\;0\leq J_{1}\leq 2 \end{array}$$ (1)

where ***NJ*** = **0** is the steady-state constraint with ***N*** as stoichiometric matrix and ***J*** as flux vector (or flux pathway). Simple metabolic models can be optimized by hand, but linear programming is required for the solution of any realistic genome-scale model. FBA optimization confirmed that, for this set of capacity constraints, maximization of our objective function gives *J*_18_ = 1.

#### Characterization of the optimal solution space for a metabolic model with reversible reactions

Several flux pathways maximize the objective *J*_18_ = 1, i.e. our FBA model is underdetermined. We can describe the optimal solution space with the Minkowski sum (see Equation (4)) in terms of three mathematical objects: linealities, rays, and vertices (Fig. 1) [29, 30]. Each of these mathematical objects relate to a topological motif in a metabolic network.

Linealities are reversible cycles or input-output pathways (boundary to boundary metabolite(s), see S1 Fig. for an example) that indicate in which flux directions the optimal solution space is unbounded. Linealities will cease to exist when we split each reversible reaction into two irreversible reactions later. The set of reactions R2, R3, R4, i.e. {R2–R4}, form a lineality (Fig. 2A). The reactions can take any value, but there must be a net flux of two through {R2–R4} that converts A into B.

Rays are irreversible (thermodynamically infeasible) cycles or input-output pathways. Our toy model does not contain a ray. If at least one of the reactions R2, R3 or R4 would have been irreversible, {R2–R4} would have been a ray (Fig. 2A).

Vertices are the corner points of the FBA polyhedron—the solution space of optimal flux distributions—nd therefore they cannot be represented as a convex combination of other optimal flux pathways (they are non-decomposable). Convex combinations of neighboring vertices form the facets—“edges”—of this polyhedron. Our toy model contains four vertices termed V1–V4 (Fig. 2B). We can use a combination of these vertices and cyclic networks to represent every optimal flux vector.

Vertices originate from combinations of alternative flux distributions in CoPE-FBA subnetworks, quickly leading to a combinatorial explosion [16]. In the optimum, these subnetworks consist of reactions with correlated flux variability and have a fixed net input-output stoichiometry of reactants and products. The toy model contains two subnetworks (Fig. 2C). In the subnetwork consisting of {R6–R10}, the alternative flux distribution {R6–R8} is anti-correlated with alternative flux distribution {R9–R10} and the reactions within these sets are positively correlated with each other (S2 Fig.). Both sets of reactions have an identical input-output relationship: D + ADP → H + ATP. We can multiply the number of alternative flux distributions through each subnetwork to obtain the total number of vertices; both subnetworks of the toy model have two alternative flux distributions—the lower and the upper branch—which gives a total of four vertices (Fig. 2B).

#### The disadvantages of metabolic models with reversible reactions

The representation of the network in its current form—where reversible reactions are not split into two irreversible reactions—complicates the characterization of the optimal solution space; not all non-decomposable optimal pathways are vertices and the set of vertices is not unique. This set of vertices corresponds to the minimal generating set of the optimal solution space, a well-known concept in EFM analysis [31]. For our toy model, we enumerated all (optimal-yield) EFMs to illustrate the difference between the set of vertices we enumerated with CoPE-FBA and the set of non-decomposable flux pathways in the optimum.

**Fig 3:**
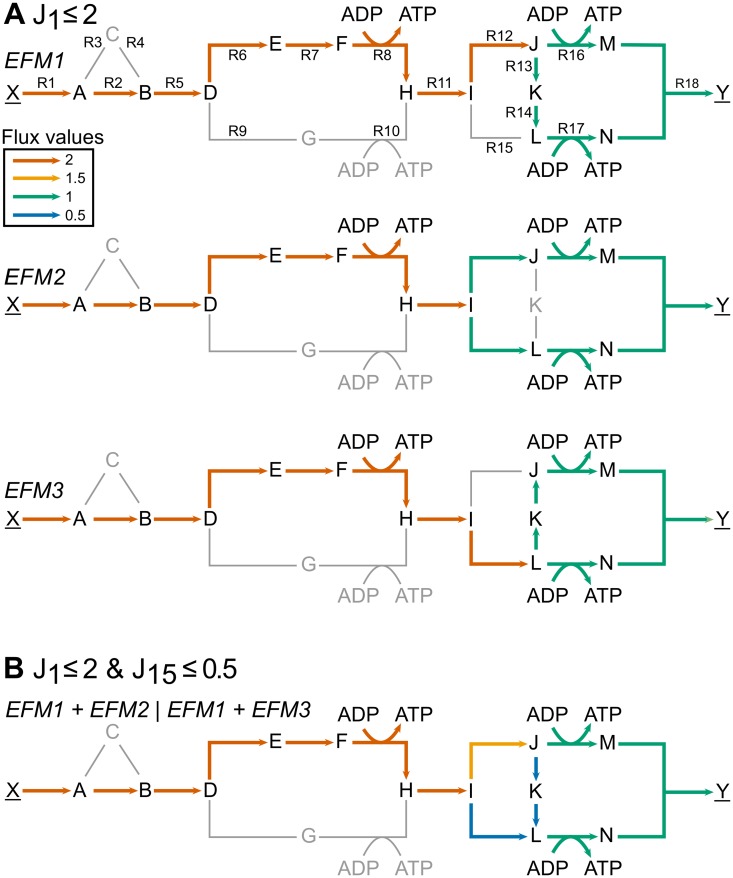
Vertices correspond to optimal-yield EFMs or convex combinations of those EFMs. Vertices correspond to optimal-yield EFMs (A) if they are restricted by one flux constraint and to a convex combination of EFMs if they are restricted by more than one flux constraint (B). Colors represent different flux values (red = 2, orange = 1.5, green = 1, and blue = 0.5). (A) visualization of EFM1, EFM2, and EFM3 (out of the twelve optimal-yield EFMs normalized to *J*_18_ = 1). Both EFM1 and EFM3 have a corresponding vertex with and without splitting, whereas EFM2 has only with splitting a corresponding vertex. (B) taking a convex combination of EFM1 and EFM2 or EFM1 and EFM3 (panel A) corresponds to a vertex when the constraints are *J*_1_ ≤ 2 and *J*_15_ ≤ 0.5.

If there is only one upper bound (as in the toy model), all non-decomposable optimal pathways are instances of the optimal-yield EFMs. EFM analysis showed that our toy model has thirteen EFMs of which twelve are operational modes that produce Y with an optimal yield (see also S3 Fig. for a more detailed analysis). Thus, for only one third of the optimal-yield EFMs, there exists a corresponding vertex (S3B Fig.). The remaining eight EFMs do not have corresponding vertices, because: (i) two non-decomposable optimal pathways are a convex combination of different vertices; e.g. 1/2 V1 (EFM1) + 1/2 V2 (EFM3) = EFM2 (Fig. 3A) and (ii) six more optimal pathways are a linear combination of vertices (or of the two convex combinations) and the lineality (with flux through {R3, R4} rather than {R2}). Because of these additional non-decomposable optimal pathways, we cannot find all the possible pathways that optimize a (secondary) objective directly from the vertex representation—we shall return to this later.

Metabolic models usually contain many sub-optimal modes—modes with a lower yield—or non-operational modes—modes that cannot produce any objective flux. Our toy network was designed to not contain suboptimal operational modes. The non-operational mode is the lineality shown in Fig. 2A.

The EFM analysis further showed that we can describe the optimal solution space with different sets of vertices. For instance, CoPE-FBA gave that {R2} was part of each vertex and {R3, R4} was only part of a lineality. We can reverse this situation by making {R2} part of the lineality and {R3, R4} part of each vertex, hence this decomposition is not unique.

#### Reversible-reaction splitting yields all non-decomposable flux pathways in the optimum

**Fig 4:**
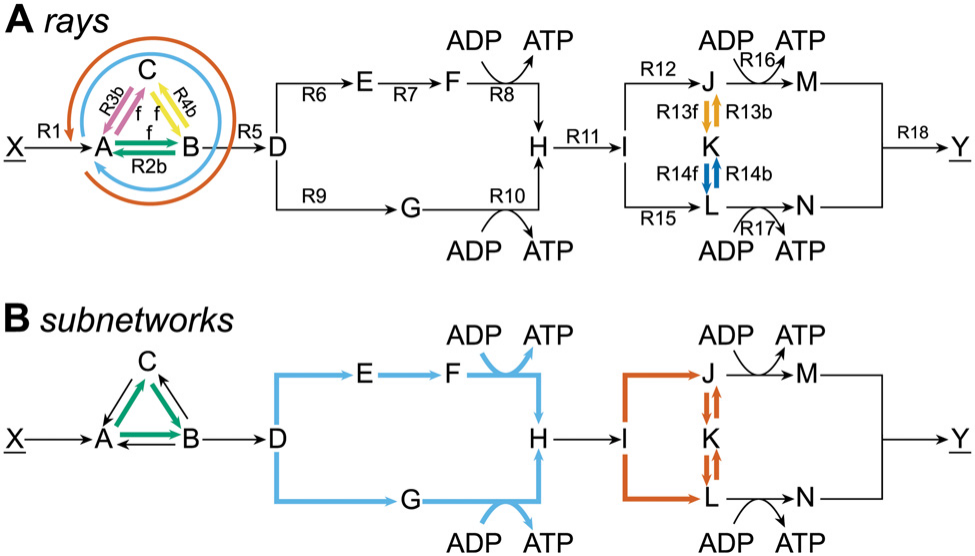
Reversible-reaction splitting guarantees finding all non-decomposable flux pathways in the optimum. Metabolites (capital letters) are converted by irreversible reactions to achieve the conversion of X to Y (underlined metabolites are boundary species). Split reversible reactions are denoted as R3f and R3b. We maximized the flux through R18 with FBA, subject to the steady-state constraint and *J*_1_ ≤ 2. The optimal solution space is now characterized by seven rays (A) and twelve vertices which originate from three CoPE-FBA subnetworks (B). (A) the five split reversible reactions and {R2–R4} in forward and backward direction form together seven rays. (B) three subnetworks give rise to twelve vertices (2×2×3). The third subnetwork (red) now has a third alternative flux distribution {R12, R15} which was without reversible-reaction splitting a convex combination of the other two flux distributions, {R12, R13f, R14f} and {R15, R13b, R14b}.

To solve the issues with uniqueness and completeness, we exploited an existing technique in the field of EFM analysis: Splitting reversible reactions into separate forward and backward reactions [31, 32]. In the split model, the vertices are instances of exactly the non-decomposable pathways in the optimal state. Since all reactions are irreversible after the splitting procedure, linealities do not exist anymore. The split toy model contains seven rays, because each split reversible reaction forms an additional ray and two more rays from {R2–R4} in forward and backward direction (see Fig. 4A). After splitting, rays no longer signify thermodynamically-infeasible irreversible cycles. Each forward and backward reaction together forms a new EFM, but the set of optimal-yield EFMs is identical before and after splitting (S3A and S3D Fig.), which was also proven by Gagneur and Klamt [33].

For our toy model, each optimal-yield EFM now has a corresponding vertex (S3E Fig.), i.e. the vertices lie on their corresponding EFMs. This means that our toy model contains now twelve rather than four vertices. To explain the difference between the set of vertices with and without splitting, we first focus on {R2–R4}. With splitting, the model contains vertices with both {R2} and {R3, R4}, while without splitting vertices only contain either {R2} or {R3, R4}. The variability in reactions {R2–R4} causes the number of vertices to double (2 × 2 × 2 vs. 2 × 2), because {R2–R4} is also a CoPE-FBA subnetwork (Fig. 4B) in the split model. The second difference originates from the CoPE-FBA subnetwork described by {R12–R15}. The flux distribution through {R12, R15} (see also EFM2 Fig. 3A) cannot be obtained via a convex combination of alternative flux distributions and is, therefore, now also a vertex. This difference causes the number of vertices to further increase from eight to twelve (2×2×3 vs. 2×2×2).

#### Additional non-zero flux constraints cause a dissimilarity between optimal-yield EFMs and vertices

After reversible-reaction splitting, the set of optimal-yield EFMs corresponds to the set of vertices if there is only a *single* non-zero restricting flux constraint (for proof see S1 Text). With more constraints this is not necessarily the case, because EFMs are based on stoichiometry and thermodynamics alone, while vertices also depend on flux constraints. We illustrate this by discussing examples of different types of flux constraints on the sets optimal-yield EFMs and vertices: setting a flux to zero, adding a restricting constraint, and adding a demanding constraint.

Setting a flux to zero (e.g. an anaerobic growth condition) effectively removes a reaction from the system. The set of optimal-yield EFMs still corresponds to the set of vertices (of course all pathways using the removed flux, e.g. oxygen uptake, are absent). As an example, removing R15 from our toy network would result in the same set of four optimal-yield EFMs and vertices.

We illustrate the effect of adding an additional restricting non-zero flux constraint (e.g. an upper bound on oxygen uptake) in our toy model with: *J*_15_ ≤ 0.5. With this constraint, *J*_18_ = 1 cannot be achieved with EFMs that include reaction R15 (e.g. EFM2 and EFM3 shown in Fig. 3A). The corresponding vertices are infeasible, because vertices are only defined in the optimal space (see also S3C and S3F Fig. for a more detailed analysis). The corner points of the new optimal solution space are now described by a different set of vertices, i.e. still feasible vertices and vertices that arose after adding the second constraint. Each newly introduced vertex arose from an infeasible vertex and a neighboring feasible vertex. An example of such a vertex is given in Fig. 3B, which corresponds to a convex combination of EFM1 and EFM2 or EFM1 and EFM3. In this example, the number of corner points of the optimal solution space decreased after adding the second flux constraint, but this is not a general outcome.

Demanding a flux through a reaction that decreases the substrate-product yield (e.g. ATP maintenance reaction) yields different vertices. An optimal-yield EFM through the demand reaction is then added to each vertex. The number of vertices increases if multiple optimal-yield EFMs coexist through the flux demanding reaction (which will then form another CoPE-FBA subnetwork).

#### Secondary optimization collapses the optimal solution space

In this section, we demonstrate how secondary optimization simplifies after reversible-reaction splitting and that secondary optimization reduces the solution space to only one or a few vertices. As a secondary optimization objective, we used minimization of the number of active fluxes—hereafter pathway length *P*_*L*_; see Equation (8). Mathematically, we can write this secondary optimization as follows:

$$\begin{array}{ccc} & & \text{Minimize}\;P_{L}\\ & & \text{subject}\;\text{to,}\\ & & \;\;\;\;\;N\;J=0\\ & & \;\;\;\;\;J_{18}=Z_{obj}=1\\ & & \;\;\;\;\;-\infty \leq J_{r}\leq \infty \;\;\;\;\;\;J_{r}\in \text{reversible}\;\text{reactions}\\ & & \;\;\;\;\;\;0\leq J_{i}\leq \infty \;\;\;\;\;\;\;\;\;\;J_{i}\in \text{irreversible}\;\text{reactions}\\ & & \;\;\;\;\;\;0\leq J_{1}\leq 2 \end{array}$$ (2)

in which we set the output flux of the toy model to its maximal value obtained with the previous FBA, shown in Equation (1). We used mixed-integer linear programming to select the flux pathway with the minimal *P*_*L*_ from the optimal solution space for one (*J*_1_ ≤ 2) and two (*J*_1_ ≤ 2, *J*_15_ ≤ 0.5) restricting flux constraints, which gave a minimal *P*_*L*_ of 11 and 12, respectively. Next, we determined the *P*_*L*_ for each optimal-yield EFM and vertex of the “non-split” and “split” model (see S4 Fig. for more details).

Without splitting, the vertices are instances of a subset of all possible non-decomposable pathways (EFMs) in the optimal state. Therefore, for the optimization of secondary objectives in the *non-split* model, we cannot focus solely on the vertices. We have to take into account the whole optimal solution space—the Minkowski sum of vertices, rays, and linealities (for details see Methods)—which is cumbersome.

Analyzing the effect of linealities and rays is counterintuitive because both linealities and rays represent cycles that catalyze no net conversion. This makes them independent from the chosen growth medium and objective function. However, without splitting, linealities and/or rays can influence secondary objectives when they share reactions with one or more vertices. In this case we can construct non-decomposable optimal flux pathways that are not vertices by taking, for instance, a linear combination of a vertex with a connected lineality. An example is the lineality described by {R2–R4} (Fig. 2A). Adding {R2, R3, -R4} to one of the four vertices gives rise to a new optimal flux pathway; one with more active reactions.

Taking a convex combination of vertices shortens *P*_*L*_ when more active reactions become inactive than vice versa. A reaction becomes inactive when it goes in different directions with the same flux in alternative flux distributions. When *J*_1_ ≤ 2 is the only constraint, a convex combination of alternative flux distributions in {R12–R15} (Fig. 2C) shortens *P*_*L*_ by one reaction: Specifically, R13 and R14 carry flux in both alternative flux distributions whereas in different directions. This analysis shows that the minimal *P*_*L*_ is a convex combination of vertices V3 and V4 (Fig. 2B). This optimal pathway becomes infeasible when we add the second flux constraint *J*_15_ ≤ 0.5; then, vertex V4 minimizes *P*_*L*_.

With splitting, we immediately obtain all possible non-decomposable pathways in the optimal state; no convex combination turns active reactions inactive, because all fluxes are positive. The shortest optimal flux pathway is always a vertex (and corresponds to an optimal-yield EFM if it is restricted by only one non-zero flux constraint). Theoretically, multiple shortest vertices can co-exist. The fact that only a vertex or several vertices optimize the secondary objective is a specific result for pathway length minimization (see also S5 Fig.). For instance, the optimal solution space after minimization of the sum of absolute fluxes as secondary objective (Equation (10)) can consist of a line or a plane, besides (multiple) single point(s).

#### Concluding remarks about reversible-reaction splitting

Reversible-reaction splitting has many advantages for the characterization of the optimal solution space. We first summarize those advantages before we set out to analyze a genome-scale stoichiometric model. Splitting of the reversible reactions leads to:

1. The vertices discovered with CoPE-FBA are all possible non-decomposable pathways in the optimal state. For the analyses of optimal flux pathways, we can, therefore, focus solely on vertices. These vertices can be compactly described by a set of subnetworks that describe all the variability in non-decomposable optimal flux pathways.
2. A unique characterization of the optimal solution space.
3. Secondary optimization yields an optimal solution space consisting of one or multiple vertices.
4. Rays no longer signify thermodynamically-infeasible irreversible cycles.

Typically, splitting yields many more vertices and rays (each split reversible reaction forms an additional ray). We identified three different mechanisms that contribute to the increase in vertices: (i) splitting can yield additional CoPE-FBA subnetworks that originate from rays or linealities with a input-output relationship different from zero. An example is the lineality given by {R02–R04} (Fig. 2A) that is a subnetwork with splitting (Fig. 4B). (ii) optimal flux pathways that are convex combinations of vertices before splitting become vertices after splitting. We encountered such a case in the toy model where the convex combination of vertices V3 and V4 resulted in an additional vertex after splitting. (iii) rays or linealities connected to CoPE-FBA subnetworks give rise to additional vertices. Imagine for the toy network, for instance, a reversible reaction that converts metabolite F into metabolite E (the reverse of reaction R7). Before splitting, R7 and this newly introduced reaction form a ray. After splitting, an additional vertex exists through this newly introduced reaction.

Enumeration of many more vertices requires more computational power, hence we developed a much more efficient method, CoPE-FBA 2.0, for enumeration of the optimal solution space of both toy and genome-scale models, which is described in detail in the Methods section. The enumeration requires now minutes to hours rather than days to weeks to complete.

### A real life example: *Escherichia coli* growing on glucose

We analyzed the realistic genome-scale stoichiometric model iAF1260 of *Escherichia coli* (*E. coli*) metabolism [34]. By modifying the oxygen uptake constraint, we constructed three different FBA models of iAF1260 that depict aerobic, aerobic restricted, and anaerobic growth (for details see Methods). We set maximization of biomass production rate as the objective function. For these growth conditions, general CoPE-FBA results for both the model with and without reversible-reaction splitting are shown in Table 1. With splitting we found for each growth condition many more vertices (up to 120 × 10^6^). Since we also used an ATP maintenance demand constraint of 8.39 *mmol* *gDW* ^−1^ *h* ^−1^, our vertices are not instances of EFMs. Without this constraint, all vertices in both the aerobic and anaerobic growth condition are instances of their corresponding EFMs (not shown). We found only a few CoPE-FBA subnetworks which together completely reveal the variability in the vertices. Most reactions are inactive after optimizing the objective function (S1 Table). In the remainder of this section, we use the results obtained from the model with splitting because this yielded all non-decomposable flux pathways in the optimum.

**Table 1:** Characterization of the optimal solution space with and without reversible-reaction splitting.

Model	Toy model		*E. coli* iAF1260 model	
Reversible-reaction splitting	No	Yes	No	Yes
Growth condition			Aerobic	
Total reactions	12	23	2374	3226
Rays	0	7	26	604
Linealities	1	0	1	0
Vertices	4	12	839.808	120.932.352
Subnetworks	2	3	6	9
Model	*E. coli* iAF1260 model		*E. coli* iAF1260 model	
Reversible-reaction splitting	No	Yes	No	Yes
Growth condition	Aerobic restricted		Anaerobic	
Total reactions	2374	3226	2374	3226
Rays	25	602	25	602
Linealities	1	0	1	0
Vertices	1.679.616	40.310.784	31104	1.492.992
Subnetworks	4	4	6	8

The optimal solution space is typically characterized by many vertices and a few rays and linealities. Vertices originate from a combinatorial explosion of only a few CoPE-FBA subnetworks. Reversible-reaction splitting yields a unique characterization of this optimal solution space, but typically many more vertices (and rays) exist. With reversible-reaction splitting, there is one additional ray in the aerobic growth condition which concerns an irreversible cycle of oxygen.

#### Gaussian and multimodal distributions of vertices after secondary optimization

**Fig 5:**
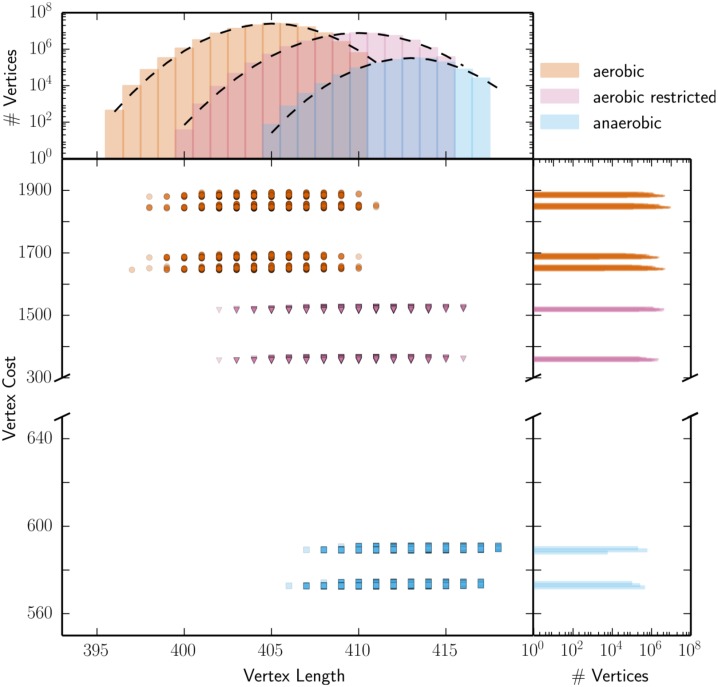
*E.coli* vertex cost follows a multimodal distribution. For three growth conditions—aerobic (red, circle), aerobic restricted (purple, triangle), and anaerobic (blue, square)—we analyzed the vertex cost (*P*_*C*_) and vertex length (*P*_*L*_) of each vertex. Each dot in the main panel represents a vertex with a specific cost and length. Our results indicate that for *E.coli* the vertex length follows approximately a Gaussian-shaped distribution (dashed lines are Gaussian distributions with sample mean and sample standard deviation). Vertex cost follows a multimodal distribution; vertices are clustered in distinct groups with a specific cost. Due to file size limitations we only show a subset (10.000) of vertices for all conditions in the scatter plot.

We studied the distributions of objective values of a secondary optimization over the vertices obtained in the first optimization. For each vertex, we determined the pathway length *P*_*L*_, pathway sum of absolute fluxes *P*_*J*_, and pathway cost *P*_*C*_ (see Methods). Similar to work done by Shlomi et al. [10], our protein cost definition was solely based on enzyme-synthesis cost. For instance, we did not take the protein lifetimes into account. Ignoring protein lifetimes implies that *P*_*J*_ and *P*_*C*_ are closely related; *P*_*C*_ is taking *P*_*J*_ multiplied with a protein cost for each individual reaction. In Fig. 5, we thus only show the results for *P*_*L*_ and *P*_*C*_. Initially, we intuitively expected many vertices with intermediate *P*_*L*_, *P*_*J*_, and *P*_*C*_, and few with relatively low or high *P*_*L*_, *P*_*J*_, and *P*_*C*_. In other words, we expected a Gaussian-shaped distribution for both *P*_*L*_, *P*_*J*_, and *P*_*C*_. As expected, the *P*_*L*_ was indeed Gaussian-shaped distributed for all tested growth conditions. This is illustrated by the dashed black lines in the top panel of Fig. 5 which correspond to a Gaussian distribution where we used the sample mean and standard deviation as input.

In contrast, *P*_*C*_ was clustered into distinct groups, i.e. a multimodal distribution. An accurate determination of pathway cost is a challenge and we hypothesized that a different cost function could show a different distribution. Therefore, we investigated four different definitions of protein cost: minimum, maximum, average, and equal (i.e. sum of absolute fluxes; *P*_*J*_). When multiple proteins were associated to a particular reaction via an OR rule, the minimum, maximum or average was taken (for more details see Methods). In all cases, we found a multimodal distribution. Nonetheless, we did find an effect of the cost function; taking the “minimal” cost function typically resulted in the largest difference between both clusters, while taking the “equal” cost function typically resulted in the smallest difference between both clusters (S2 Table, S6 Fig.). These results show that for explaining the multimodal distribution of vertex cost, the effect of fluxes was much more important than the effect of protein costs.

We already explained that enumeration of a model with reversible reactions does generally not result in a unique characterization of the optimal solution space. Hence, different subsets of all non-decomposable flux pathways in the optimum can be found. To demonstrate the possible differences, we enumerated the *E.coli* iAF1260 model including reversible reactions for the same conditions of which results are shown in S7 Fig. Comparison with Fig. 5 shows that during aerobic growth conditions only two rather than four clusters were found.

#### CoPE-FBA subnetworks explain differences in secondary optimization

CoPE-FBA subnetwork analysis revealed the shape of the distributions of the length, sum of absolute fluxes, and cost of vertices. Several different CoPE-FBA subnetworks contributed to the total length difference and within the majority of these subnetworks we found alternative flux distributions with different lengths; the length distribution within the subnetworks were uniform or already Gaussian in shape. It is, therefore, not possible to reconstruct many vertices with a short or long vertex length, which explains the Gaussian-shaped distribution of vertex length.

Alternatively, one (aerobic restricted and anaerobic cases) or two subnetworks (aerobic case) explain the main differences in *P*_*J*_ and *P*_*C*_. Within these subnetworks, we found two distinct modes—a relatively cheap and a relatively expensive mode. While some of these subnetworks were relatively large, our results show that the main cost difference in this particular subnetwork originates from only a few metabolic reactions (S3 Table). As a consequence, we found many vertices with a relatively low *P*_*C*_ and many vertices with a relatively high *P*_*C*_—a multimodal distribution of vertex cost. In the aerobic growth conditions, the cost difference mainly emerged from using different electron acceptors for the NADH dehydrogenase; cheap pathways used ubiquinone-8 and costly pathways used menaquinone-8 and/or demethylmenaquinone-8. Interestingly, in aerobic growth conditions ubiquinone-8 is the major quinone in *E. coli* [35, 36]. In anaerobic growth conditions, the cost difference mainly emerged from exploiting a different strategy for the ATP-dependent conversion from PEP and F6P to DHAP, G3P, and PYR in main carbon metabolism (S8 Fig.).

Lastly, we studied the reduction of the solution space after secondary optimization. For the *E. coli* iAF1260 model with splitting, secondary optimization reduced the solution space to only one or a few vertices (Table 2). In case of *P*_*L*_-minimization, only vertices can be optimal solutions, since convex combinations increase the number of active reactions. Compared to minimization of *P*_*J*_ and *P*_*C*_, the solution space after minimization of *P*_*L*_ contained more vertices in all of the tested growth conditions. This was expected because *P*_*L*_ is solely based on the number of active reactions, specific flux values are not of interest. Taking these flux values into account typically results in more diverse outcomes. Hence, it is less likely to find as many vertices with a minimal *P*_*J*_. Similarly, adding different protein costs to each reactions further diversifies these outcomes. As a result, the optimal solution space for *P*_*C*_-minimization resulted in a unique flux distribution for all tested growth conditions.

**Table 2:** Secondary optimization can reduce the optimal solution space to a unique flux distribution.

	*E. coli* iAF1260 growth condition		
	Aerobic	Aerobic restricted	Anaerobic
min(*P*_*L*_)	432	36	72
min(*P*_*J*_)	24	24	24
min(*P*_*c*_)	1	1	1

For the *E.coli* iAF1260 genome-scale model, secondary optimization reduced the optimal solution space from 1–120 million vertices to about 1–432 vertices. When we minimize pathway cost (*P*_*c*_) a unique optimal flux distribution exists, but many suboptimal flux distributions do exist (Fig. 5).

## Discussion

The recently developed computational method, CoPE-FBA (Comprehensive Polyhedra Enumeration Flux Balance Analysis) [16], offers the premise of a simplified biological understanding of the optimal solution space of metabolic network models; a kind of understanding which is not possible with other popular methods such as Flux Variability Analysis [14] and Flux Coupling Analysis [25]. We further developed this method: Rather than enumerating the minimal generating set, we used reversible-reaction splitting [31, 32] to enumerate all non-decomposable flux pathways in the optimum. This allows us to focus solely on the vertices for the analysis of optimal flux pathways.

Enumerating all non-decomposable flux pathways in the optimum is a very demanding task compared to enumerating only a (small) subset of these flux pathways; especially for CoPE-FBA as presented by Kelk et al [16]. Therefore, we also developed an efficient computational method, CoPE-FBA 2.0, for the (unique) characterization of the optimal solution space. We can now characterize the optimal solution space in the order of minutes for most (bacterial) genome-scale models on just an ordinary computer. CoPE-FBA 2.0 is efficient because it first determines the subnetworks and subsequently enumerates the vertices for each subnetwork (see Methods for more details). To illustrate this, the 120 ⋅ 10^6^ vertices enumerated for *E. coli* under aerobic growth conditions originate from eight subnetworks with respectively 6, 3, 5184, 3, 2, 54, 2, 2 vertices. This means that while we determined in total only 5256 vertices (the sum), we actually enumerated 120.932.352 vertices (the multiplication) within 15 minutes on an ordinary computer.

The further development of CoPE-FBA facilitated in achieving a better understanding of how optimal flux pathways resulting from FBA arise out of EFMs, use of constraints, and optimality conditions. We recall that the vertices correspond to optimal-yield EFMs if there is *only* a single restricting flux constraint. Both restricting and demanding flux constraints modify the (optimal) solution space. Typically, the optimization problem remains underdetermined and an optimal solution space will continue to exist. We can get a unique solution by adding additional constraints that concern all flux values in the model (e.g. protein cost constraints). Then, the optimal state is an instance of an optimal-yield EFM if there is only a single restricting flux constraint. Alternatively, the optimal state corresponds to a convex combination of optimal-yield EFMs. For this reason, we can also use CoPE-FBA 2.0 to quickly enumerate all optimal-yield EFMs, which can be useful because enumerating the complete set of EFMs of a genome-scale model is a laborious undertaking [37, 38].

Other constraints that also concern all reactions, but not their flux values, such as minimal *P*_*L*_, will often lead to optimal solution spaces. While these objectives have been used frequently to find more realistic FBA outcomes [18–22], we showed that for both minimization of *P*_*L*_ and *P*_*C*_, optimal solution spaces continue to exist (Table 2). This result shows that we should be careful drawing conclusions from predicted flux distributions after using a secondary objective. Using CoPE-FBA with only irreversible reactions allows for a straightforward identification of the origin of the remaining solution space. Specifically, these solution spaces originate from identically favorable pathways through CoPE-FBA subnetworks. Similarly, we can use these CoPE-FBA subnetworks to directly explain the differences after secondary optimization, as we showed for the multimodal distributions of vertex cost.

In this research, we further demonstrated the use of CoPE-FBA 2.0 for the *E. coli* iAF1260 genome-scale model by determining *P*_*L*_ and *P*_*C*_ for each enumerated vertex for different growth conditions. We found Gaussian-shaped and multimodal distributions for *P*_*L*_ and *P*_*C*_, respectively. These results can be further used to deduce a hypothesis of the selection pressure if we know the flux distribution. If the objective of *E. coli* would be to minimize *P*_*C*_ (or *P*_*J*_), we would not expect *E. coli* to exploit the unique optimal solution since the difference between this optimal solution and many suboptimal solutions is almost negligible. We do, however, expect *E. coli* to exploit the “cheap” reactions that cause the bi- or multimodal distribution of vertex cost. Interestingly, in aerobic conditions, our analysis predicted that all cheap pathways exploit ubiquinone-8 which is also the major electron acceptor in *E. coli* under these conditions [35, 36]. If the objective of *E. coli* would be to minimize *P*_*L*_, many different flux vectors give rise to an optimal or near-optimal solution. The multitude of optimal solutions hinders the construction of a hypothesis about *P*_*L*_ from individual reactions. In future research we see as possible application of our method, finding the minimal flux distance between alternative optimal flux vectors in different conditions, to answer questions about how species can adapt to changing conditions.

In conclusion, we present a better understanding of the principles of the optimal solution space and an efficient method to enumerate all non-decomposable flux pathways in this state. This paves the way to answer biological questions about the flexibility of organisms while growing at optimal states in a fast and straightforward manner. This work, therefore, contributes to reaching a topological understanding of metabolic functionality in the optimum in terms of metabolic flux pathways. In the future, the development of graphical maps [39] can further simplify the analysis by allowing for straightforward visualization and inspection of these metabolic flux pathways.

## Methods

### Flux balance analysis

For a metabolic network of *m* metabolites and *r* reactions, the *m* × *r* stoichiometric coefficients are often represented in the stoichiometric matrix ***N***. The stoichiometric coefficient *n*_*ij*_ is positive if metabolite *i* is net produced in reaction *j*, negative if metabolite *i* is net consumed in reaction *j*, and otherwise zero. The representation of a metabolic network in a stoichiometric model is particularly useful for constraint-based modeling techniques like Flux Balance Analysis (FBA). By using a linear programming approach, FBA can optimize (maximize or minimize) an objective function subject to the steady-state constraint, thermodynamic constraints, and capacity constraints:

$$\begin{array}{ccc} & & \text{Maximize}\;\text{or}\;\text{Minimize}\;Z_{obj}=c^{T}J\\ & & \text{subject}\;\text{to,}\\ & & \;\;\;\;\;N\;J=0\\ & & \;\;\;\;\;J^{\min}\leq J\leq J^{\max} \end{array}$$ (3)

Here, ***c*** is a vector of coefficients that represent the contribution of each flux in vector ***J*** to the objective function *Z*_*obj*_. Next, ***NJ*** = **0** is the steady-state constraint. Finally, ***J*** ^***min***^ and ***J*** ^***max***^ specify the minimal and maximal flux values for each reaction. In addition to providing a unique optimal outcome of the objective function, FBA provides a corresponding optimal flux distribution ***J*** ^***opt***^. Most FBA models are underdetermined systems, and therefore, many corresponding ***J*** ^***opt***^ exist. For more details about FBA, we refer to Orth et al. [12].

### Minkowski sum

The Minkowski sum given in Equation (4) provides the description of any ***J*** ^***opt***^ in terms of vertices, rays, and linealities [16, 29].

$$\begin{array}{c} J^{\mathrm{opt}}=\sum_{k=1}^{s}\alpha_{k}\varphi_{k}+\sum_{k=1}^{t}\beta_{k}\varphi_{k}+\sum_{k=1}^{u}\gamma_{k}\psi_{k} \end{array}$$ (4)

Here, the vectors ***φ***_***k***_, ***φ***_***k***_, and ***ψ***_***k***_ represent the vertices, rays, and linealities, respectively. Additionally, *s*, *t*, and *u* represent the upper boundaries of the sum functions indicating the number of vertices, rays, and linealities, respectively. Furthermore, *α*_*k*_, *β*_*k*_, and *γ*_*k*_ represent the weighting coefficient that satisfy the following constraints: $\sum_{k=1}^{s}\alpha_{k}=1$, *α*_*k*_ ≥ 0, *β*_*k*_ ≥ 0, and *γ*_*k*_ can take any value. In words, vertices can be summed by a convex combination, rays can be summed as a conical combination, and linealities can be summed as a linear combination.

This Minkowski sum alters (Equation (5)) once we split each reversible reaction into two irreversible reactions, because linealities do not exist anymore.

$$\begin{array}{c} J^{\mathrm{opt}}=\sum_{k=1}^{s}\alpha_{k}\varphi_{k}+\sum_{k=1}^{t}\beta_{k}\varphi_{k} \end{array}$$ (5)

Each split reversible reaction fulfills all conditions for a ray, thus many more rays are found when we split each reversible reaction. Each of these additional rays is also an EFM and an extreme pathway which are considered irrelevant because they only reformulate reversibility [32, 40].

### CoPE-FBA subnetworks and F-modules

The set of vertices yields a flux space without futile cycles. CoPE-FBA subnetworks are defined within this flux space and have a fixed input-output relationship, which we can write mathematically as:

$$\begin{array}{c} N_{A}J_{A}=d\neq 0 \end{array}$$ (6)

where ***A*** is a vector of reactions that form the subnetwork [41]. Subsequently, ***N***_***A***_ and ***J***_***A***_ are the stoichiometric matrix and the flux vector of the subnetwork, and ***d*** is the fixed input-output relationship of the subnetwork. We can also calculate subnetworks (modules) in a flux space with futile cycles. These subnetworks are called F-modules and can be determined via FluxModules [42]. We can distinguish two types of F-modules:

- F-modules essential for optimality, i.e. ***d*** ≠ **0**
- F-modules not essential for optimality, i.e. ***d*** = **0**

F-modules not essential for optimality are rays or linealities not connected to optimal flux pathways. An F-module essential for optimality can be a CoPE-FBA subnetwork, a ray or lineality connected to optimal flux pathways, or a combination of those.

### CoPE-FBA 2.0 pipeline

Kelk et al. 2012 [16] developed the CoPE-FBA pipeline developed to characterize the optimal solution space in terms of vertices, rays, and linealities. Enumeration of genome-scale models without reversible-reaction splitting can take already several days with this computational method. We developed a new pipeline, CoPE-FBA 2.0, to make this enumeration less memory and CPU intensive. First, we preprocessed the model as also described by Kelk et al. [16]. Then, we executed the following steps:

1. **Determine F-modules and extract the fixed network**. We used a Python implementation of FluxModules to quickly determine the F-modules.
2. **Determine *d* for each F-module**. To circumvent numerical issues we used rational FBA (QSopt_EX version 2.5.0 [43]) to determine ***d***. FBA output was also used to set the values of the fixed network.
3. **Reconstruct F-module models**. For each F-module we reconstructed a model that consisted only of the reactions and metabolites of the F-module. We added input and output reactions to fix **d** of each F-module essential for optimality in the optimal solution space. Dummy species were added both the input and output reaction to guarantee use of both reactions.
4. **Perform CoPE-FBA as described in Kelk et al. 2012 [16] for each F-module**. Enumeration on each F-module essential for optimality yielded all vertices. F-modules not essential for optimality were enumerated to determine the total number of rays.
5. **Reconstruct network vertices.** We merged fixed parts and the enumerated vertices for all subnetworks.

Enumerating the optimal solution space via CoPE-FBA 2.0 took minutes to hours rather than days to weeks for the original pipeline developed by Kelk et al. 2012 [16]. The CoPE-FBA 2.0 pipeline and all data files used during these study are available for download from http://memesa-tools.sf.net.

### Rank test

In the constructed *E.coli* subnetworks, the input-output relationship was the only constraint. Consequently, each enumerated subnetwork vertex should correspond to an optimal-yield EFM of the subnetwork. We successfully used the rank test [44] to show that each enumerated “subnetwork vertex” ***v*** is an instance of an (optimal-yield) EFM. First, we determined the zero indices of ***v***. Second, from the stoichiometric matrix ***N*** of the subnetwork we eliminated all columns with a zero index in ***v*** to create a submatrix ***N***_***nz***_. Third, we used single value decomposition to determine the rank of ***N***_***nz***_. Last, we used the rank–nullity theorem to determine its nullity (Equation (7)), the dimension of the right nullspace which should be one if ***v*** is an EFM.

$$\begin{array}{c} nullity\;N_{\mathrm{nz}}=1 \end{array}$$ (7)

Theoretically, enumerated vertices of subnetworks do not have to be instances of optimal-yield EFMs, because additional restricting flux constraints can be located inside these subnetworks. In addition, even if all vertices of all subnetworks were instances of optimal-yield EFMs, vertices describing the optimal pathways through the complete network do not have to correspond to EFMs. This is only true if there is one restricting non-zero flux constraint and no demanding flux constraints.

### Secondary objectives

As a secondary objective, we used, in addition to pathway length (*P*_*L*_) and pathway sum of absolute fluxes (*P*_*J*_), also pathway cost (*P*_*C*_)—a proxy for the minimization of the ATP utilization in protein synthesis—to reduce the size of the solution space.

$$\begin{array}{ccc} P_{L} & = & \left|\{J_{j}:J_{j}\neq 0\}\right| \end{array}$$ (8)

$$\begin{array}{ccc} P_{J} & = & \sum_{j=1}^{r}\left|J_{j}\right| \end{array}$$ (9)

$$\begin{array}{ccc} P_{C} & = & \sum_{j=1}^{r}c_{j}\left|J_{j}\right| \end{array}$$ (10)

The *P*_*L*_ is identical to the number of flux carrying reactions, while the *P*_*C*_ is identical to the sum of absolute flux values multiplied with *c*_*j*_, the protein cost for each individual reaction. This cost is the scaled length of the proteins that were associated to this reaction, which we used as a proxy for all costs. In other words, *c*_*j*_ < 1 when the associated protein length is smaller than average and vice versa. We set *c*_*j*_ = 1 when no information about associated proteins was available. Because multiple proteins can be associated to a particular reaction via AND and OR rules, different definitions of *c*_*j*_ were used: maximum, average, minimum, and equal. An AND rule corresponds to taking the sum of protein lengths, while an OR rule corresponds to taking the maximum, average, or minimum. Using equal cost is identical to minimizing the sum of absolute fluxes, a widely-used secondary objective. Taking the maximum, average, minimum, or equal definition of *c*_*j*_ did not effect the interpretation of our results.

### Genome-scale models

The aerobic restricted version (maximum O_2_ uptake was 18.5 *mmol* *gDW* ^−1^ *h* ^−1^) of iAF1260 was obtained from the BiGG database [45]. Maximum glucose uptake was set to 12.77 *mmol* *gDW* ^−1^ *h* ^−1^ and we modified the bounds on the O_2_ uptake reaction to create specific aerobic (no constraint on O_2_ uptake) and anaerobic (exchange of O_2_ set to zero) conditions. In all cases, the model required an ATP maintenance flux of 8.39 *mmol* *gDW* ^−1^ *h* ^−1^. The model was edited and prepared for enumeration using PySCeS CBMPy [46, 47]. All models are provided as Supplementary Dataset S1. Optimization of secondary objectives (minimization of *P*_*L*_, *P*_*J*_, and *P*_*C*_) was also done with PySCeS CBMPy. We used a mixed-integer linear program to minimize *P*_*L*_ and a linear program to minimize *P*_*J*_ and *P*_*C*_.

## Supporting Information

S1 Dataset A zip-file containing SBML files encoding the metabolic networks used in this manuscript. (ZIP)

S1 Fig Examples of rays in the *E.coli* iAF1260 genome-scale metabolic model. (PDF)

S2 Fig Subnetworks consist of reactions with correlated flux variability. (PDF)

S3 Fig Vertices correspond to optimal-yield EFMs if they are restricted by a single limiting flux constraint. (PDF)

S4 Fig Minimization of *P*_*L*_ yields one or more vertices with reversible-reaction splitting. (PDF)

S5 Fig Example of the effect of splitting reversible reactions. (PDF)

S6 Fig Multimodal vertex cost distributions are consistent for different definitions of cost. (PDF)

S7 Fig Different cost distributions after enumerating the model with reversible reactions. (PDF)

S8 Fig The cost explaining module in the anaerobic growth conditions. (PDF)

S1 Text Proof that the polytope of a single-constraint FBA has vertices that lie on EFMs. (PDF)

S1 Table Most reactions carry no flux in the optimal solution. (PDF)

S2 Table Flux is the main contributor to the observed cost difference between vertices. (PDF)

S3 Table Cost explaining reactions for the three different growth conditions of *E.coli* iAF1260. (PDF)

## References

[1] Edwards JS, Palsson BØ (1999) Systems properties of the Haemophilus influenzae Rd metabolic genotype. J Biol Chem 274: 17410–17416. 10.1074/jbc.274.25.17410 10364169

[2] Edwards JS, Palsson BØ (2000) The Escherichia coli MG1655 in silico metabolic genotype: its definition, characteristics, and capabilities. Proc Natl Acad Sci USA 97: 5528–5533. 10.1073/pnas.97.10.5528 10805808 PMC25862

[3] Thiele I, Palsson BØ (2010) A protocol for generating a high-quality genome-scale metabolic reconstruction. Nat Protoc 5: 93–121. 10.1038/nprot.2009.203 20057383 PMC3125167

[4] Henry CS, DeJongh M, Best AA, Frybarger PM, Linsay B, et al (2010) High-throughput generation, optimization and analysis of genome-scale metabolic models. Nat Biotechnol 28: 977–982. 10.1038/nbt.1672 20802497

[5] Price ND, Reed JL, Palsson BØ (2004) Genome-scale models of microbial cells: evaluating the consequences of constraints. Nat Rev Microbiol 2: 886–897. 10.1038/nrmicro1023 15494745

[6] Oberhardt MA, Palsson BØ, Papin JA (2009) Applications of genome-scale metabolic reconstructions. Mol Syst Biol 5: 320 10.1038/msb.2009.77 19888215 PMC2795471

[7] Teusink B, Smid EJ (2006) Modelling strategies for the industrial exploitation of lactic acid bacteria. Nat Rev Microbiol 4: 46–56. 10.1038/nrmicro1319 16357860

[8] Nogales J, Gudmundsson S, Thiele I (2013) Toward systems metabolic engineering in cyanobacteria: opportunities and bottlenecks. Bioengineered 4: 158–163. 10.4161/bioe.22792 23138691 PMC3669157

[9] Raman K, Rajagopalan P, Chandra N (2005) Flux balance analysis of mycolic acid pathway: targets for anti-tubercular drugs. PLoS Comput Biol 1: e46 10.1371/journal.pcbi.0010046 16261191 PMC1246807

[10] Shlomi T, Benyamini T, Gottlieb E, Sharan R, Ruppin E (2011) Genome-scale metabolic modeling elucidates the role of proliferative adaptation in causing the Warburg effect. PLoS Comput Biol 7: e1002018 10.1371/journal.pcbi.1002018 21423717 PMC3053319

[11] Lee JM, Gianchandani EP, Papin JA (2006) Flux balance analysis in the era of metabolomics. Brief Bioinformatics 7: 140–150. 10.1093/bib/bbl007 16772264

[12] Orth JD, Thiele I, Palsson BØ (2010) What is flux balance analysis? Nat Biotechnol 28: 245–248. 10.1038/nbt.1614 20212490 PMC3108565

[13] Feist AM, Palsson BØ (2010) The biomass objective function. Curr Opin Microbiol 13: 344–349. 10.1016/j.mib.2010.03.003 20430689 PMC2912156

[14] Mahadevan R, Schilling CH (2003) The effects of alternate optimal solutions in constraint-based genome-scale metabolic models. Metab Eng 5: 264–276. 10.1016/j.ymben.2003.09.002 14642354

[15] Reed JL, Palsson BØ (2004) Genome-scale in silico models of E. coli have multiple equivalent phenotypic states: assessment of correlated reaction subsets that comprise network states. Genome Res 14: 1797–1805. 10.1101/gr.2546004 15342562 PMC515326

[16] Kelk SM, Olivier BG, Stougie L, Bruggeman FJ (2012) Optimal flux spaces of genome-scale stoichiometric models are determined by a few subnetworks. Sci Rep 2: 580 10.1038/srep00580 22896812 PMC3419370

[17] Stelling J, Klamt S, Bettenbrock K, Schuster S, Gilles ED (2002) Metabolic network structure determines key aspects of functionality and regulation. Nature 420: 190–193. 10.1038/nature01166 12432396

[18] Blank LM, Kuepfer L, Sauer U (2005) Large-scale 13C-flux analysis reveals mechanistic principles of metabolic network robustness to null mutations in yeast. Genome Biol 6: R49 10.1186/gb-2005-6-6-r49 15960801 PMC1175969

[19] Snitkin ES, Dudley AM, Janse DM, Wong K, Church GM, et al (2008) Model-driven analysis of experimentally determined growth phenotypes for 465 yeast gene deletion mutants under 16 different conditions. Genome Biol 9: R140 10.1186/gb-2008-9-9-r140 18808699 PMC2592718

[20] Grafahrend-Belau E, Schreiber F, Koschutzki D, Junker BH (2009) Flux balance analysis of barley seeds: a computational approach to study systemic properties of central metabolism. Plant Physiol 149: 585–598. 10.1104/pp.108.129635 18987214 PMC2613719

[21] Mazumdar V, Snitkin ES, Amar S, Segrè D (2009) Metabolic network model of a human oral pathogen. J Bacteriol 191: 74–90. 10.1128/JB.01123-08 18931137 PMC2612419

[22] Collins SB, Reznik E, Segrè D (2012) Temporal expression-based analysis of metabolism. PLoS Comput Biol 8: e1002781 10.1371/journal.pcbi.1002781 23209390 PMC3510039

[23] Schuetz R, Kuepfer L, Sauer U (2007) Systematic evaluation of objective functions for predicting intracellular fluxes in *Escherichia coli* . Mol Syst Biol 3: 119 10.1038/msb4100162 17625511 PMC1949037

[24] Holzhütter HG (2004) The principle of flux minimization and its application to estimate stationary fluxes in metabolic networks. Eur J Biochem 271: 2905–2922. 10.1111/j.1432-1033.2004.04213.x 15233787

[25] Burgard AP, Nikolaev EV, Schilling CH, Maranas CD (2004) Flux coupling analysis of genome-scale metabolic network reconstructions. Genome Res 14: 301–312. 10.1101/gr.1926504 14718379 PMC327106

[26] Wiback SJ, Famili I, Greenberg HJ, Palsson BØ (2004) Monte Carlo sampling can be used to determine the size and shape of the steady-state flux space. J Theor Biol 228: 437–447. 10.1016/j.jtbi.2004.02.006 15178193

[27] Bilu Y, Shlomi T, Barkai N, Ruppin E (2006) Conservation of expression and sequence of metabolic genes is reflected by activity across metabolic states. PLoS Comput Biol 2: e106 10.1371/journal.pcbi.0020106 16933982 PMC1550272

[28] Bordel S, Agren R, Nielsen J (2010) Sampling the solution space in genome-scale metabolic networks reveals transcriptional regulation in key enzymes. PLoS Comput Biol 6: e1000859 10.1371/journal.pcbi.1000859 20657658 PMC2904763

[29] Grötschel M, Lovász L, Schrijver A (1988) Geometric algorithms and combinatorial optimization. Springer-Verlag.

[30] Maarleveld TR, Khandelwal RA, Olivier BG, Teusink B, Bruggeman FJ (2013) Basic concepts and principles of stoichiometric modeling of metabolic networks. Biotechnol J 8: 997–1008. 10.1002/biot.201200291 23893965 PMC4671265

[31] Wagner C, Urbanczik R (2005) The geometry of the flux cone of a metabolic network. Biophys J 89: 3837–3845. 10.1529/biophysj.104.055129 16183876 PMC1366950

[32] Klamt S, Stelling J (2003) Two approaches for metabolic pathway analysis? Trends Biotechnol 21: 64–69. 10.1016/S0167-7799(02)00034-3 12573854

[33] Gagneur J, Klamt S (2004) Computation of elementary modes: a unifying framework and the new binary approach. BMC Bioinformatics 5: 175 10.1186/1471-2105-5-175 15527509 PMC544875

[34] Feist AM, Henry CS, Reed JL, Krummenacker M, Joyce AR, et al (2007) A genome-scale metabolic reconstruction for *Escherichia coli* K-12 MG1655 that accounts for 1260 ORFs and thermodynamic information. Mol Syst Biol 3: 121 10.1038/msb4100155 17593909 PMC1911197

[35] Unden G, Bongaerts J (1997) Alternative respiratory pathways of *Escherichia coli*: energetics and transcriptional regulation in response to electron acceptors. Biochim Biophys Acta 1320: 217–234. 10.1016/S0005-2728(97)00034-0 9230919

[36] Bekker M, Alexeeva S, Laan W, Sawers G, Teixeira de Mattos J, et al (2010) The ArcBA two-component system of *Escherichia coli* is regulated by the redox state of both the ubiquinone and the menaquinone pool. J Bacteriol 192: 746–754. 10.1128/JB.01156-09 19933363 PMC2812447

[37] Klamt S, Stelling J (2002) Combinatorial complexity of pathway analysis in metabolic networks. Mol Biol Rep 29: 233–236. 10.1023/A:1020394300385 12241063

[38] Acuna V, Marchetti-Spaccamela A, Sagot MF, Stougie L (2010) A note on the complexity of finding and enumerating elementary modes. BioSystems 99: 210–214. 10.1016/j.biosystems.2009.11.004 19962421

[39] Maarleveld TR, Boele J, Bruggeman FJ, Teusink B (2014) A data integration and visualization resource for the metabolic network of *Synechocystis* sp. PCC 6803. Plant Physiol 164: 1111–1121. 10.1104/pp.113.224394 24402049 PMC3938606

[40] Papin JA, Price ND, Palsson BØ (2002) Extreme pathway lengths and reaction participation in genome-scale metabolic networks. Genome Res 12: 1889–1900. 10.1101/gr.327702 12466293 PMC187577

[41] Müller AC, Bockmayr A (2014) Flux modules in metabolic networks. J Math Biol 69: 1151–1179. 10.1007/s00285-013-0731-1 24141488

[42] Müller A, Bruggeman F, Olivier B, Stougie L (2014) Fast flux module detection using matroid theory In: Research in Computational Molecular Biology, Springer International Publishing, volume 8394 of *Lecture Notes in Computer Science*. pp. 192–206. 10.1089/cmb.2014.0141 25565150

[43] Applegate DL, Cook W, Dash S, Espinoza DG (2007) Exact solutions to linear programming problems. Operations Research Letters 35: 693–699. 10.1016/j.orl.2006.12.010

[44] Klamt S, Gagneur J, Von Kamp A (2005) Algorithmic approaches for computing elementary modes in large biochemical reaction networks. Syst Biol (Stevenage) 152: 249–255. 10.1049/ip-syb:20050035 16986267

[45] Schellenberger J, Park J, Conrad T, Palsson BØ (2010) BiGG: a Biochemical Genetic and Genomic knowledgebase of large scale metabolic reconstructions. BMC Bioinformatics 11: 213 10.1186/1471-2105-11-213 20426874 PMC2874806

[46] Olivier BG, Rohwer JM, Hofmeyr JH (2005) Modelling cellular systems with PySCeS. Bioinformatics 21: 560–561. 10.1093/bioinformatics/bti046 15454409

[47] Olivier, BG (2011). PySCeS CBMPy: Constraint Based Modelling in Python. Http://cbmpy.sourceforge.net.

