# Supplementary material for: Interplay between Constraints, Objectives, and Optimality for Genome-Scale Stoichiometric Models
Source: PLoS Comput Biol. 2015 Apr 7;11(4):e1004166. doi: 10.1371/journal.pcbi.1004166 (PMC4388735; doi:10.1371/journal.pcbi.1004166)
Supplement: S1 Fig — (PDF) [file pcbi.1004166.s002.pdf]

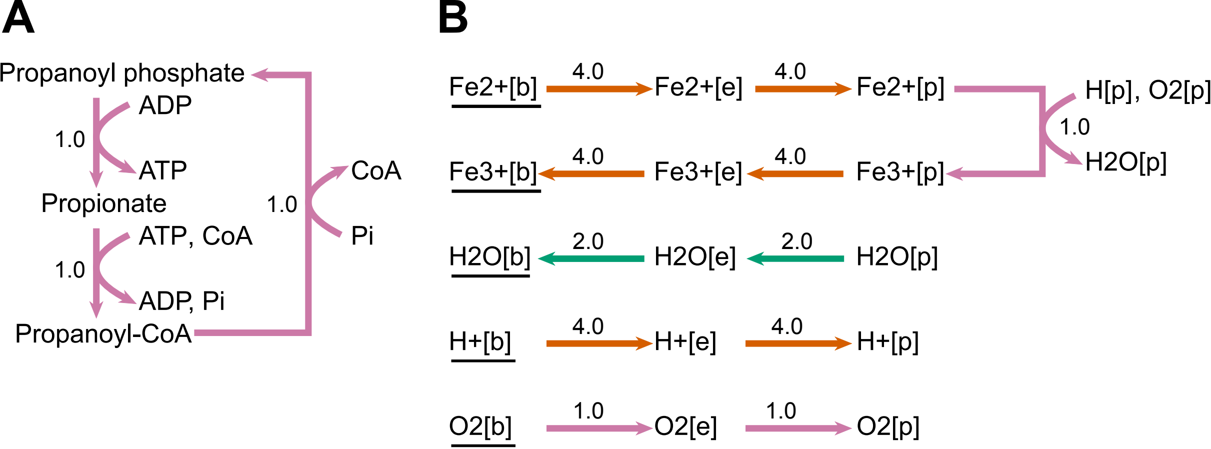

Figure S1. Examples of rays in the *E.coli* iAF1260 genome-scale metabolic model. Linealities and rays are typically cycles with no not conversion, but they can also also represent uptake of boundary compound(s) which are then converted/transported and then excreted again. Boundary metabolites are fixed and are therefore not part of the stoichiometric matrix. For both types, we found examples in the *E. coli* iAF1260 genome-scale metabolic network which are shown in (A) and (B), respectively. For the ray shown in (B), the net conversion of boundary metabolites is 4 Fe^{2+} + 4 H^{+} + $O_2 \rightarrow$ 4 Fe^{3+} + 2 H_2O. These boundary metabolites are not part of the stoichiometric matrix, thus there is no net conversion in the model.
