# Supplementary material for: Interplay between Constraints, Objectives, and Optimality for Genome-Scale Stoichiometric Models
Source: PLoS Comput Biol. 2015 Apr 7;11(4):e1004166. doi: 10.1371/journal.pcbi.1004166 (PMC4388735; doi:10.1371/journal.pcbi.1004166)
Supplement: S2 Fig — (PDF) [file pcbi.1004166.s003.pdf]

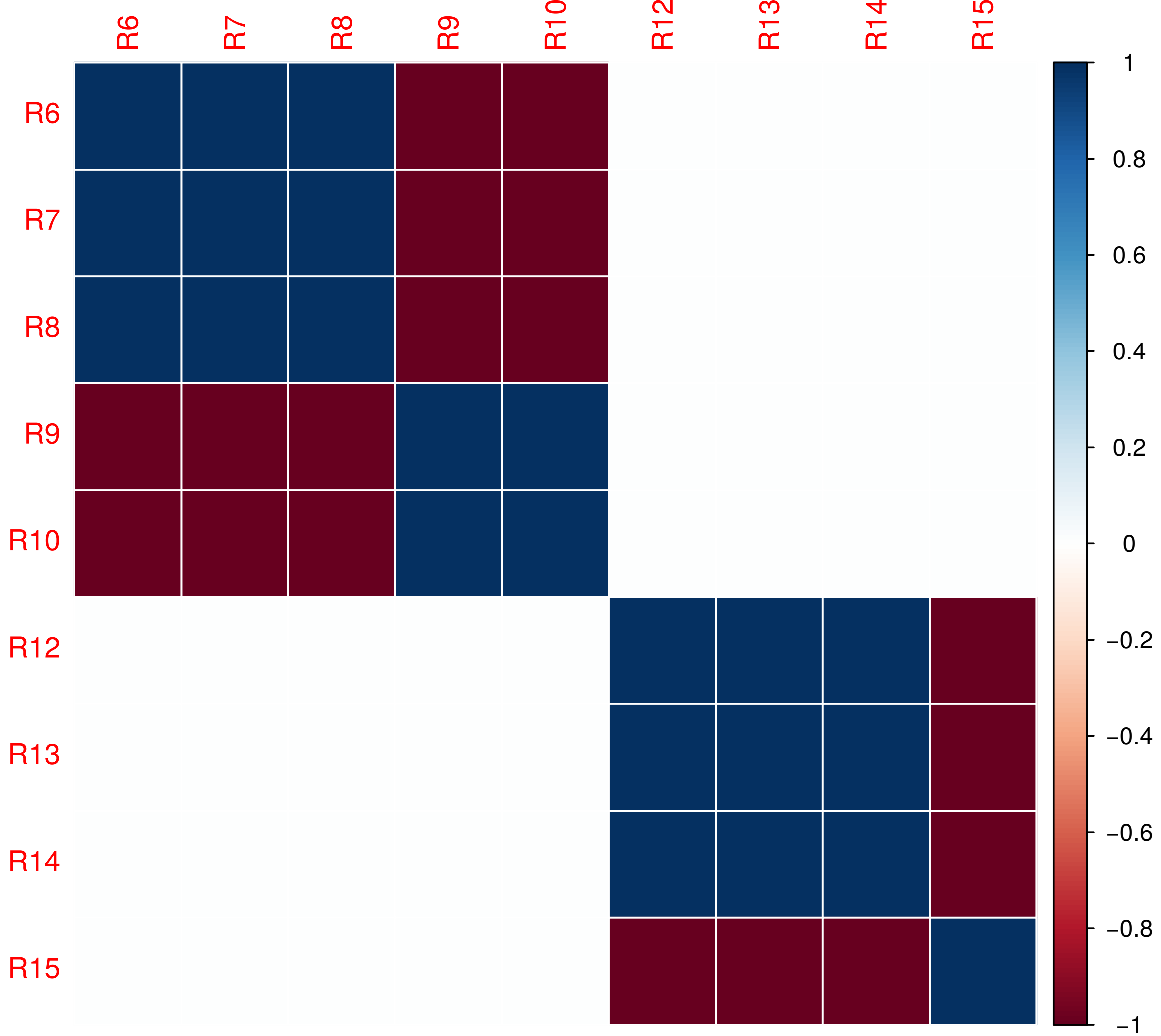


**Figure S2. Subnetworks consist of reactions with correlated flux variability**. We performed vertex correlation analysis on the four vertices (Figure 2B) of our metabolic toy network without reversible-reaction splitting. This figure shows the Pearson correlation coefficients. We can see that there are two subnetworks, {R6-R10} and {R12-R15}. Reactions within the same flux distribution (e.g. R6 and R7) are positively correlated and reactions between alternative flux distributions (e.g. R6 and R10) are negatively correlated.
