# Supplementary material for: Interplay between Constraints, Objectives, and Optimality for Genome-Scale Stoichiometric Models
Source: PLoS Comput Biol. 2015 Apr 7;11(4):e1004166. doi: 10.1371/journal.pcbi.1004166 (PMC4388735; doi:10.1371/journal.pcbi.1004166)
Supplement: S3 Fig — (PDF) [file pcbi.1004166.s004.pdf]

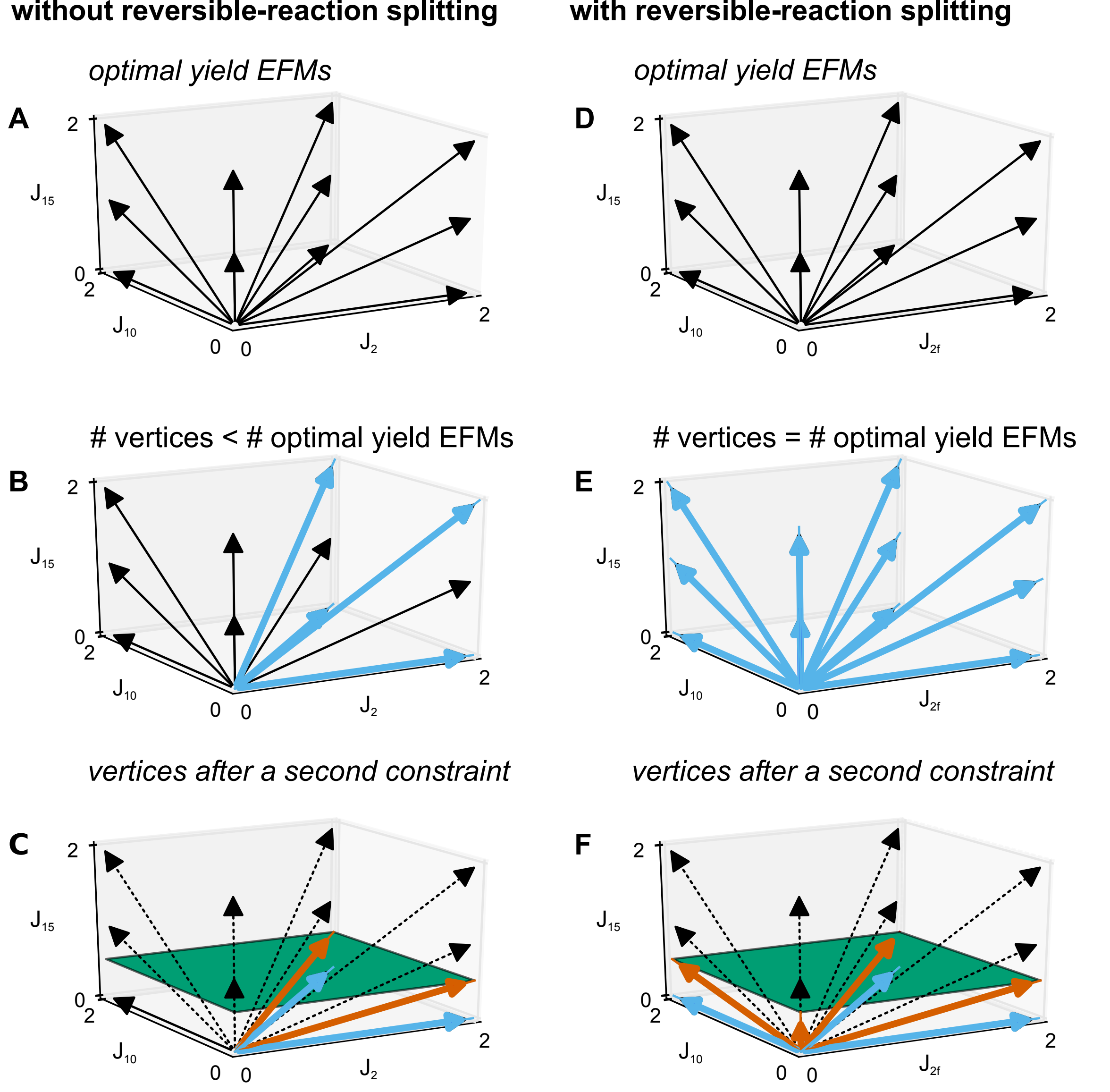

Figure S3. Vertices lie on optimal-yield EFMs if they are restricted by a single limiting flux constraint. We fixed one ($J_1 = 2$) of the four independent fluxes to create a 3D-representation of optimal-yield EFMs and vertices that span the optimal solution space. Results for the model without and with reversible-reaction splitting are shown in panels A-C and D-F, respectively. (A, D) twelve normalized optimal-yield EFMs (black arrows). Only 11 arrows are drawn since $J_2 = J_{10} = J_{15} = 0$ for one of the EFMs. (B, E) optimal-yield EFMs with corresponding vertices (blue) and optimal-yield EFMs without corresponding vertices (black) of the FBA problem presented in Equation 1 of the main text. (C, F) the (optimal) solution space shrinks after adding a second non-zero flux constraint ($J_{15} \leq 0.5$), green plane). New vertices (red) arise that are convex combinations of original EFMs/vertices. Dashed black arrows indicate EFMs that cannot achieve $J_{18} = 1$.
