# Supplementary material for: Interplay between Constraints, Objectives, and Optimality for Genome-Scale Stoichiometric Models
Source: PLoS Comput Biol. 2015 Apr 7;11(4):e1004166. doi: 10.1371/journal.pcbi.1004166 (PMC4388735; doi:10.1371/journal.pcbi.1004166)
Supplement: S4 Fig — (PDF) [file pcbi.1004166.s005.pdf]

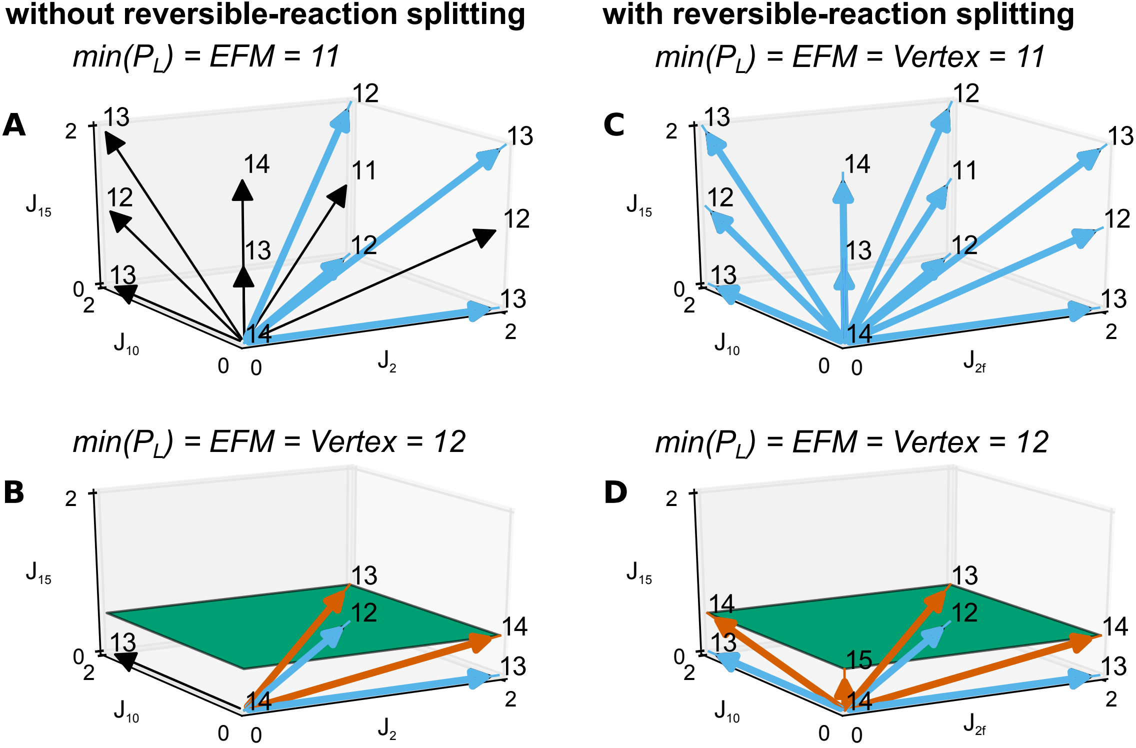

Figure S4. Minimization of pathway length (P_L) yields one or more vertices with reversible-reaction splitting. Results for the model without and with reversible-reaction splitting are shown in panels A–B and C–D, respectively. Numbers at the arrows give the P_L. For each optimal-yield EFM and vertex, we determined its P_L. See Figure S3 for an explanation of e.g. the colors used. (A, C) min(P_L) returns an optimal-yield EFM from which a corresponding vertex exists with reversible-reaction splitting. (B, D) min(P_L) returns an optimal-yield EFM from which a corresponding vertex exists with and without reversible-reaction splitting. Adding the (non-zero) second flux constraint increased min(P_L) by one. Infeasible optimal-yield EFMs which are not part of the optimal solution space are not shown.
