# Supplementary material for: Interplay between Constraints, Objectives, and Optimality for Genome-Scale Stoichiometric Models
Source: PLoS Comput Biol. 2015 Apr 7;11(4):e1004166. doi: 10.1371/journal.pcbi.1004166 (PMC4388735; doi:10.1371/journal.pcbi.1004166)
Supplement: S5 Fig — (PDF) [file pcbi.1004166.s006.pdf]

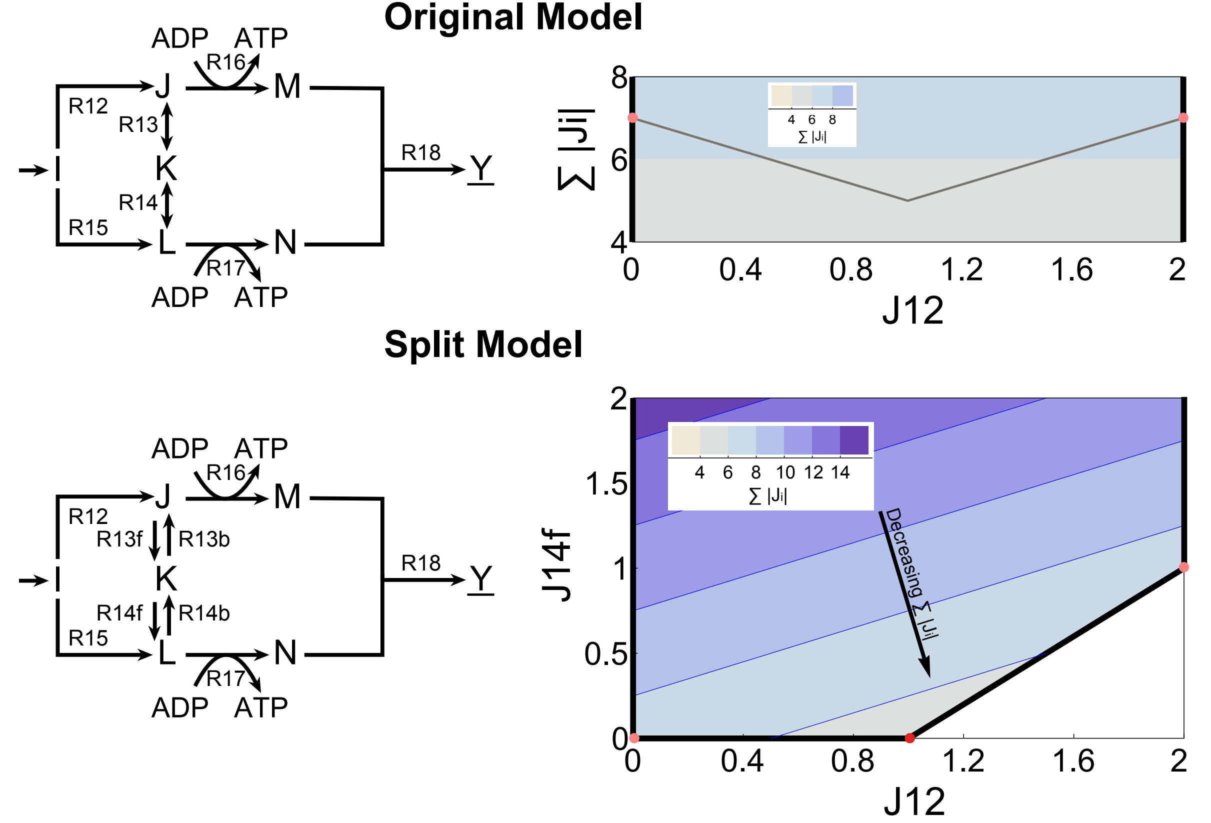

Figure S5. Example of the effect of splitting reversible reactions. We will graphically explain what happens when we first do a standard FBA and subsequently an additional minimization of the sum of absolute fluxes (P_J) in that optimum for both a network with and without reversible-reaction splitting. For this, we use a specific part of the toy network shown in Figure 2 of the main text. We first maximize J_{18} under the constraints $J_{12} + J_{15} \leq 2$ which gives $J_{18} = 1.0$. We now fix J_{18} at this rate and minimize $\sum_j |J_j|$. For the non-split network, we can express this objective in terms of J_{12} (with the steady-state assumption and $J_{18} = 1.0$) as follows: $5 + 2|J_{12} - 1.0|$ (gray line). This is not a linear function and, therefore, the minimum can lie, as in this case, between the two vertices. This is indicated in the right top graph where pink dots indicate vertices and the thick lines the boundary of the domain (which in this case is exactly the vertices). A second approach is to split all reversible reactions. This increases the possible solution space, since we can now have futile cycling with R13f and R13b and with R14f and R14b. We have drawn this new solution space, which now has an additional dimension, therefore we have shown the value of the objective $(7 - 2J_{12} + 4J_{14f})$ with a color function, in the right bottom graph (for the case that the cycling in R13 and R14 is equal; the general case is equivalent, only the solution space has one more dimension). Again the boundary of the domain is shown by the thick black lines, which now include convex combinations of vertices and linear combinations of linealities. We can see that there exists one additional vertex (red dot). Flux distributions that are not on the bottom of the domain have futile cycling. Minimization of our secondary objective is now a linear function, because all J_j are positive. The right bottom graph shows that the minimum will always be attained at a vertex and that there will be no futile cycling.
