# Supplementary material for: Interplay between Constraints, Objectives, and Optimality for Genome-Scale Stoichiometric Models
Source: PLoS Comput Biol. 2015 Apr 7;11(4):e1004166. doi: 10.1371/journal.pcbi.1004166 (PMC4388735; doi:10.1371/journal.pcbi.1004166)
Supplement: S6 Fig — (PDF) [file pcbi.1004166.s007.pdf]

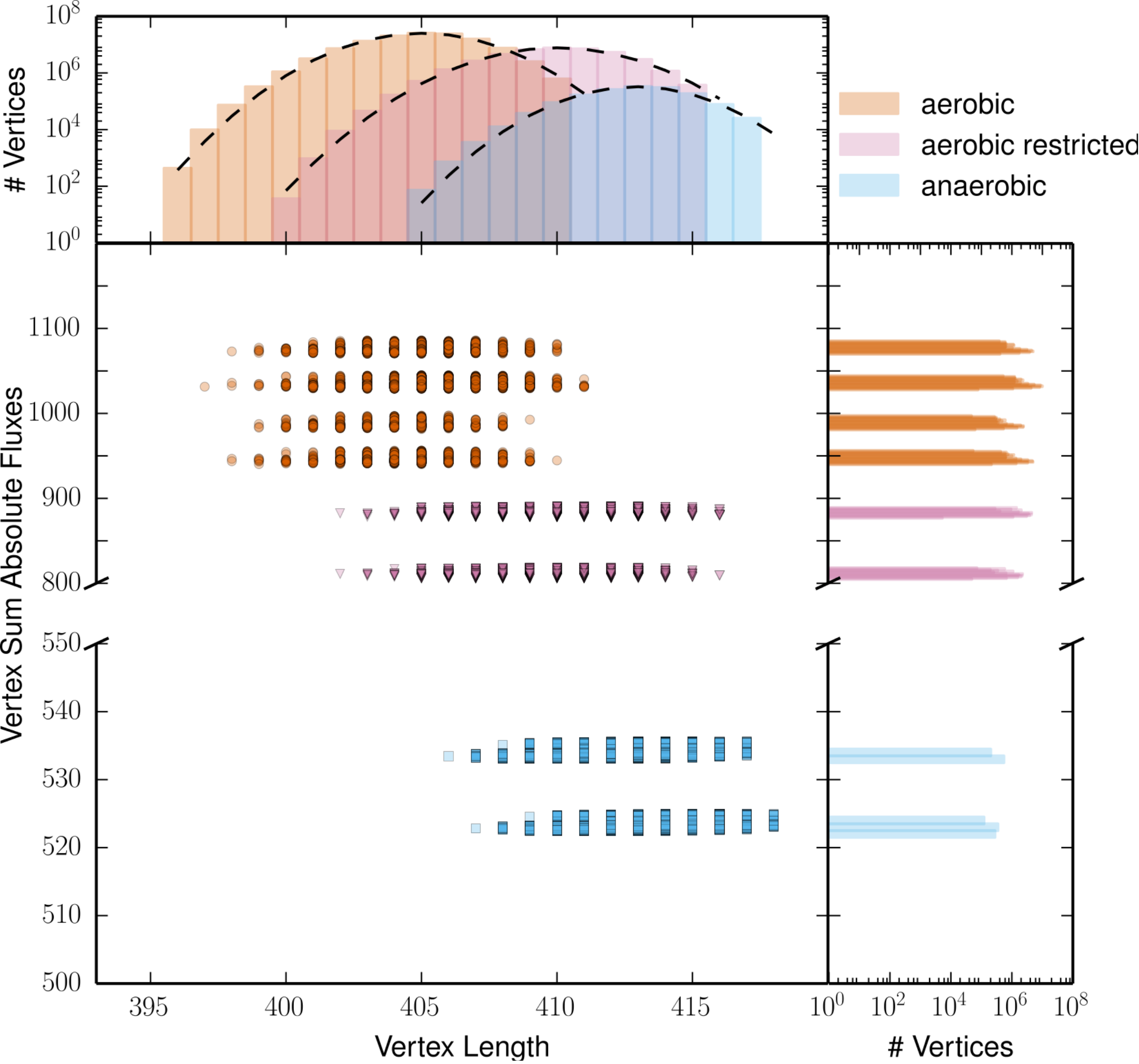

Figure S6. Multimodal vertex cost distributions are consistent for different definitions of cost. Comparison with Figure 5 of the main text shows that using the "equal" cost definition (i.e. the sum of absolute fluxes) yields a similar distribution of vertex cost. The difference between the identified clusters is less apparent in the "equal" cost definition. However, the cost definition did not alter the interpretation of the results presented in this study. Cost distributions obtained with a"min" and "average" cost definition are also similar (not shown). Note that due to file size limitations we only show a subset (10.000) of vertices for all conditions in the scatter plot.
