# Supplementary material for: Interplay between Constraints, Objectives, and Optimality for Genome-Scale Stoichiometric Models
Source: PLoS Comput Biol. 2015 Apr 7;11(4):e1004166. doi: 10.1371/journal.pcbi.1004166 (PMC4388735; doi:10.1371/journal.pcbi.1004166)
Supplement: S7 Fig — (PDF) [file pcbi.1004166.s008.pdf]

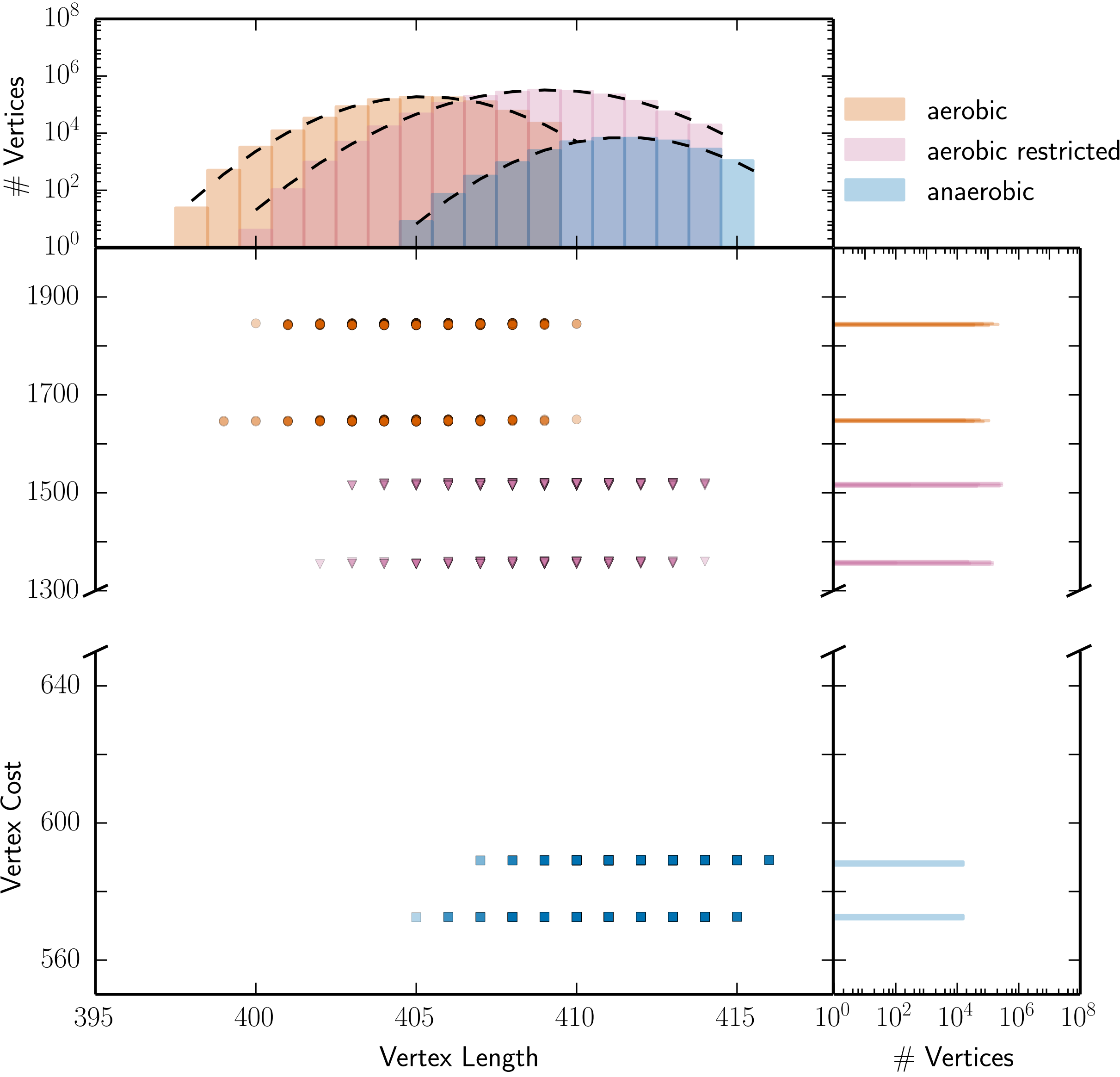

Figure S7. Different cost distributions after enumerating the model with reversible reactions. Enumeration of the optimal solution space with reversible reactions did not result in a unique characterization of this space. Different subsets of all non-decomposable optimal flux pathways can, therefore, be used to describe the optimal solution space. This can result in different properties of the set of vertices. Comparison with Figure 5 shows that especially the cost distributions are different. Here, only bimodal distributions were found while also a multimodal distribution was found by enumerating the network with reversible-reaction splitting. Note that due to file size limitations we only show a subset (10.000) of vertices for all conditions in the scatter plot.
