# Supplementary material for: Interplay between Constraints, Objectives, and Optimality for Genome-Scale Stoichiometric Models
Source: PLoS Comput Biol. 2015 Apr 7;11(4):e1004166. doi: 10.1371/journal.pcbi.1004166 (PMC4388735; doi:10.1371/journal.pcbi.1004166)
Supplement: S8 Fig — (PDF) [file pcbi.1004166.s009.pdf]

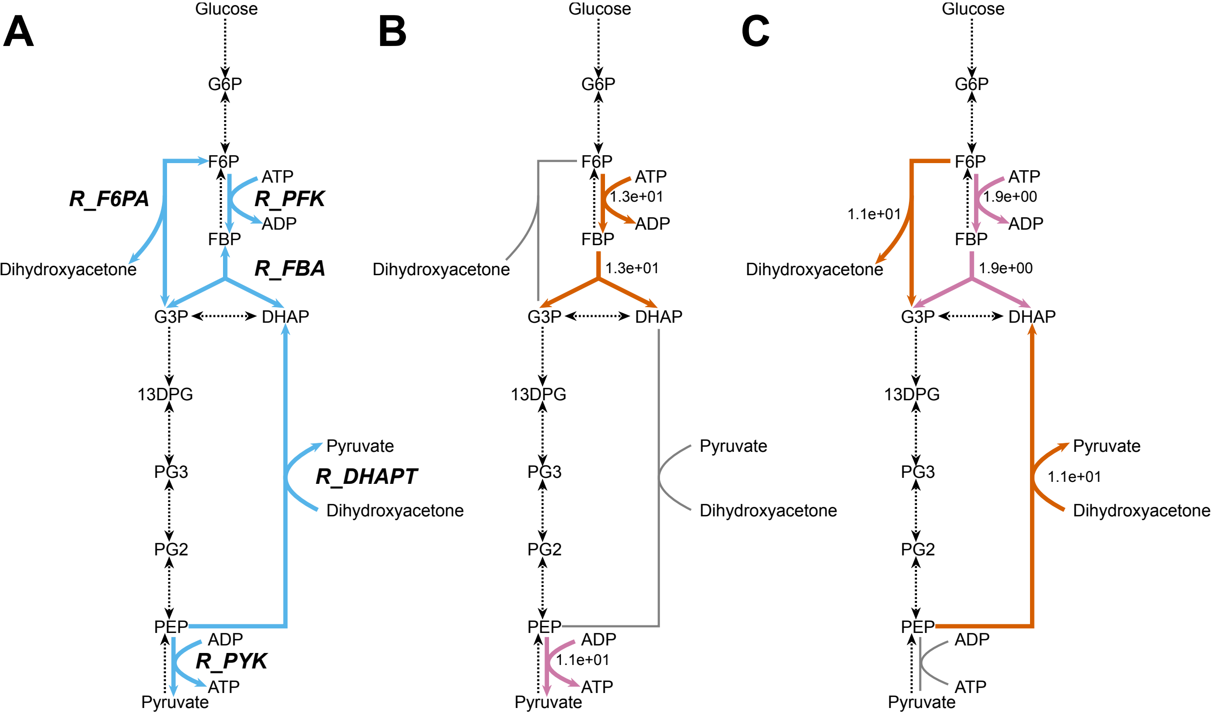

Figure S8. The cost explaining module in the anaerobic growth conditions. This module consists of five different reactions (blue) of which the identifiers are shown in bold and italics (A). Predicted normalized flux values are shown in (B) and (C) for the cheapest and costliest mode, respectively. High fluxes are marked red and low flux are marked purple. Important abbreviations: PEP (Phosphoenolpyruvate), F6P (D-Fructose 6-phosphate), DHAP (Dihydroxyacetone phosphate), G3P (Glyceraldehyde 3-phosphate), and PYR (Pyruvate).
