# Supplementary material for: Interplay between Constraints, Objectives, and Optimality for Genome-Scale Stoichiometric Models
Source: PLoS Comput Biol. 2015 Apr 7;11(4):e1004166. doi: 10.1371/journal.pcbi.1004166 (PMC4388735; doi:10.1371/journal.pcbi.1004166)
Supplement: S1 Text — (PDF) [file pcbi.1004166.s010.pdf]

Text S1: Proof

Here we describe the proof that after reversible-reaction splitting, each optimal-yield elementary flux mode (EFM) has an instance that is a vertex if there is only a *single* non-zero restricting flux constraint. This proof is analogues to the proof that the solution to the optimization of a specific flux are always extreme rays as described by Wortel et al. (2014) [1]. In this proof we use the results by Gagneur & Klamt (2004) [33] that we can decompose all reversible fluxes in a metabolic network and that the extreme rays of the cone of all steady-state solutions are the EFMs of the original network.

We want to maximize a linear objective function (e.g. biomass) given a capacity constraint on an input reaction, $J_i \leq 1$. This is the only constraint, thus we can say that $J_i = 1$ in the optimum. We can formulate this linear program (LP) as follows:

$$\max_{\boldsymbol{J}} \left\{ \boldsymbol{c}^T \times \boldsymbol{J} \middle| \boldsymbol{N}\boldsymbol{J} = \boldsymbol{0}, \boldsymbol{J}^{\boldsymbol{min}} \leq \boldsymbol{J} \leq \boldsymbol{J}^{\boldsymbol{max}},\ \underbrace{J_i = 1}_{\text{hyperplane}} \right\}. \tag{S1}$$

Here, $\boldsymbol{N}$ is the stoichiometric matrix, $\boldsymbol{c}$ is a vector of coefficients that represent the contribution of each flux in vector $\boldsymbol{J}$ to the objective function. We can simplify this LP by reversible-reaction splitting, defining all fluxes as positive. This introduces a new stoichiometry matrix $\bar{\boldsymbol{N}}$ and rate vector $\bar{\boldsymbol{J}}$,

$$\bar{\boldsymbol{J}}^{\boldsymbol{opt}} = \arg\max_{\bar{\boldsymbol{J}}} \left\{ \boldsymbol{c}^T \times \bar{\boldsymbol{J}} \middle| \underbrace{\bar{\boldsymbol{N}}\bar{\boldsymbol{J}} = \boldsymbol{0}, \bar{\boldsymbol{J}} \geq 0}_{\text{C, cone, a convex set}},\ \underbrace{J_i = 1}_{\text{hyperplane}} \right\}. \tag{S2}$$

The feasible flux space is the cone C defined in Equation (S2) (Figure S9A). C is characterized by its extreme rays. The intersection of the cone C with the plane $J_i = 1$ (Figure S9B) defines the solution space (Figure S9C),

$$P = \left\{ \boldsymbol{J} \middle| C \cap J_i = 1 \right\}. \tag{S3}$$

The extreme points of the polyhedron P are the extreme rays of C. Gagneur & Klamt (2004) proved that these extreme rays are the EFMs of the original metabolic network. Next, we maximize the linear function $\boldsymbol{c}^T \times \bar{\boldsymbol{J}}$ over P which defines the optimal solution space (Figure S9D),

$$V = \left\{ \boldsymbol{J} \middle| P \cap \boldsymbol{c}^T \bar{\boldsymbol{J}}^{\boldsymbol{opt}} \right\}. \tag{S4}$$

The corner points of V are EFMs of C (but not every EFM is a corner point of V). Since we maximize a linear function over polyhedron P, the optimum is achieved at either a corner point (vertex), an edge or a face. The objective function is often simple and includes only a single or a few fluxes (e.g. biomass production), thus the optimal solution often coincides with a face of the (many-dimensional) polyhedron (in contrast to the optimization problem in Wortel et al.). The vertices of this optimal face coincide with the vertices of the polyhedron P, and are therefore instances of EFMs.

In other words, in this proof we performed two EFM elimination steps: (i) EFMs that do not satisfy $J_i = 1$ are not part of P and (ii) EFMs that do not satisfy $\boldsymbol{c}^T \times \bar{\boldsymbol{J}}^{\boldsymbol{opt}}$ are not part of V. This means that by fixing $J_i = 1$ and by maximizing $\boldsymbol{c}^T \times \bar{\boldsymbol{J}}$ we only delete corner points of C; we do not create new corner points (as would be the case when we include additional constraints). These corner points are also defined as vertices of the optimal solution space, thus the set of vertices of V correspond to the set of optimal yield EFMs if there is only a *single* non-zero restricting flux constraint.

Supporting Information References

1. Wortel MT, Peters H, Hulshof J, Teusink B, Bruggeman FJ (2014) Metabolic states with maximal specific rate carry flux through an elementary flux mode. FEBS J 281: 1547–1555.

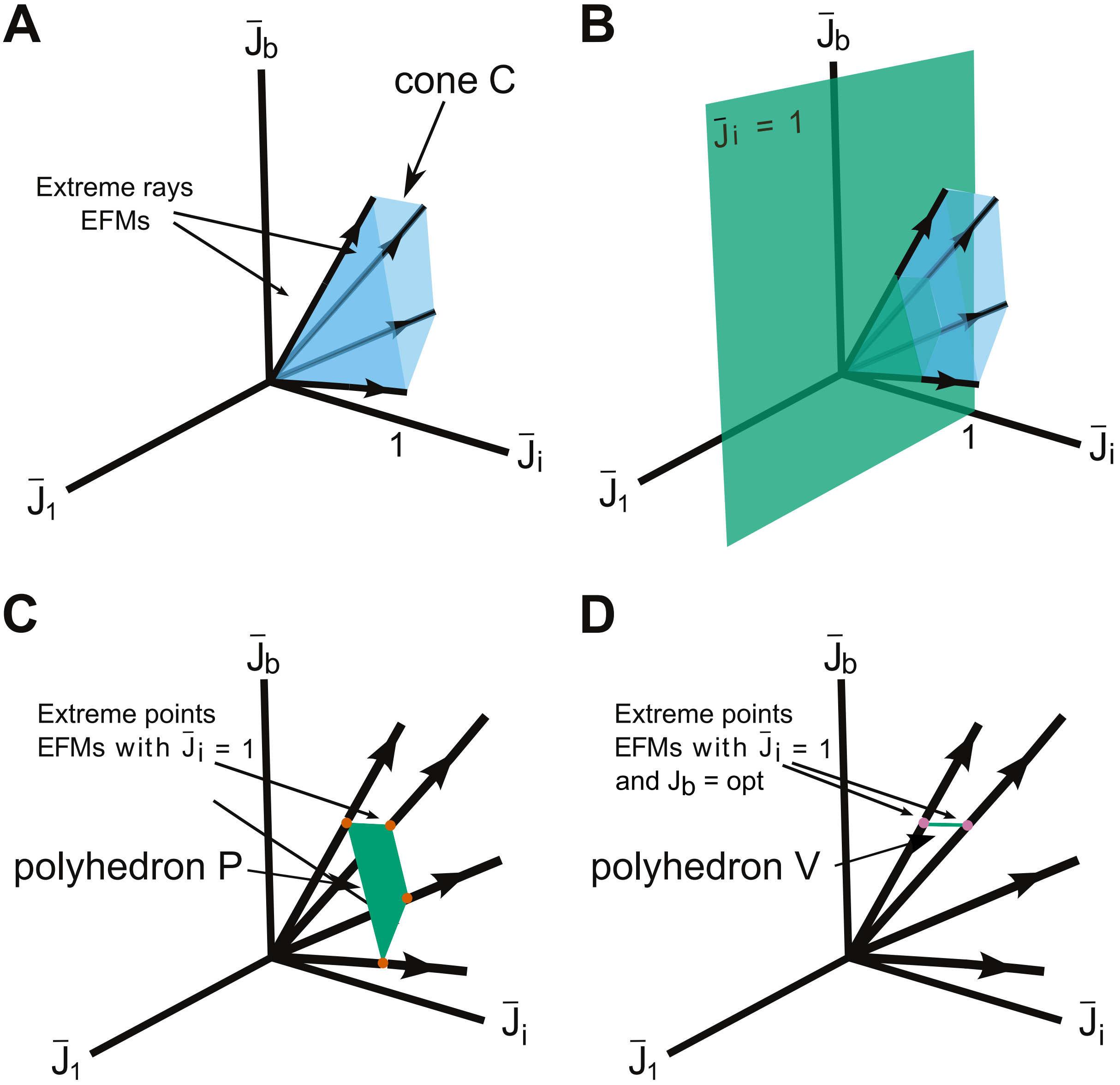

Figure S9. Schematic representation of the optimization of the biomass objective function (J_b). We used reversible-reaction splitting to make all reaction rates positive. The resulting solution space is a pointed cone C (A). Its extreme rays are the intersection of the planes with the zero vector. These extreme rays coincide with the EFMs as proven by Gagneur & Klamt (2004). Before we maximize the biomass objective function, J_b, we first fix the input reaction, $J_i = 1$ (C). This yields a polyhedron P. Finally, we maximize J_b (which is a linear function) and obtain the optimal solution space V. The corner points are vertices and optimal-yield EFMs with $J_i = 1$.
