# Supplementary material for: Interplay between Constraints, Objectives, and Optimality for Genome-Scale Stoichiometric Models
Source: PLoS Comput Biol. 2015 Apr 7;11(4):e1004166. doi: 10.1371/journal.pcbi.1004166 (PMC4388735; doi:10.1371/journal.pcbi.1004166)
Supplement: S1 Table — (PDF) [file pcbi.1004166.s011.pdf]

	E. coli iAF1260 growth condition		
Reactions	Aerobic	Aerobic restricted	Anaerobic
Zero	1904	1900	1907
Fixed non-zero	355	324	374
Variable	115	150	93
Total	2374	2374	2374

Table S1. Characterization of the optimal solution space with and without reversible-reaction splitting. For the growth conditions tested, we show that the majority of reactions is fixed in the optimum. In fact, most reactions have a zero flux in the optimal solution. Only a relatively small amount of reactions is variable after optimization of the objective function.
