# Supplementary material for: Interplay between Constraints, Objectives, and Optimality for Genome-Scale Stoichiometric Models
Source: PLoS Comput Biol. 2015 Apr 7;11(4):e1004166. doi: 10.1371/journal.pcbi.1004166 (PMC4388735; doi:10.1371/journal.pcbi.1004166)
Supplement: S2 Table — (PDF) [file pcbi.1004166.s012.pdf]

	E. coli iAF1260 growth condition		
Cost Type	Aerobic	Aerobic restricted	Anaerobic
Equal	15.5 %	10.5 %	2.5 %
Max	15.2 %	12.8 %	3.3 %
Average	15.7 %	13.4 %	3.7 %
Min	16.2 %	14.1 %	4.3 %

Table S2. Flux is the main contributor to the observed cost difference between vertices. Percentage difference between the cheapest and costliest vertex for different cost functions (equal, min, average, max; see Methods for more details). When assuming equal costs for all reactions, we effectively calculated the sum of absolute fluxes for each vertex. In this case, the percentage difference between both groups is typically smaller, but still indisputable.
