# Supplementary material for: Interplay between Constraints, Objectives, and Optimality for Genome-Scale Stoichiometric Models
Source: PLoS Comput Biol. 2015 Apr 7;11(4):e1004166. doi: 10.1371/journal.pcbi.1004166 (PMC4388735; doi:10.1371/journal.pcbi.1004166)
Supplement: S3 Table — (PDF) [file pcbi.1004166.s013.pdf]

iAF1260	ΔP_C	$\min(P_C)$	$\max(P_C)$
Aerobic	79.7 %	R_NADH16pp<br>R_FRD2, R_FRD3	R_NADH17pp,(R_NADH18pp),R_SUCDi,
	14.8 %	R_EX_h_e,R_Htex	R_EX_fe2_e,(R_EX_fe3_e),R_FE2tex (R_FE3tex)
Aerobic restricted	99.5 %	R_NADH16pp<br>R_FRD2, R_FRD3	R_NADH17pp,(R_NADH18pp), R_SUCDi,
Anaerobic	88.5 %	R_PYK, R_FBA, R_PFK	R_DHAPT,R_F6PA

Table S3. Cost explaining reactions for the three different growth conditions of *E.coli* iAF1260. Only a few reactions contribute to the differences in P_C. Here, we show the results obtained when using the maximum cost function. In aerobic conditions this cost difference is mainly explained (80-99.5%) by using a different electron carriers for the NADH dehydrogenease (ubiquinone-8 versus menaquinone-8 or demethylmenaquinone-8). Alternatively, in anaerobic conditions, the cost difference is mainly explained (88.5 %) by taking a different pathway in central carbon metabolism (Figure S6). Reactions shown in parenthesis offer alternatives, so R_NADH18pp is an alternative for R_NADH17pp.
